# Identification of Novel Endogenous Controls for qPCR Normalization in SK-BR-3 Breast Cancer Cell Line

**DOI:** 10.3390/genes12101631

**Published:** 2021-10-17

**Authors:** Nityanand Jain, Ingrida Mitre, Dina Nitisa, Valdis Pirsko, Inese Cakstina-Dzerve

**Affiliations:** Laboratory of Molecular Genetics, Institute of Oncology, Faculty of Medicine, Riga Stradiņš University, Dzirciema Street 16, LV-1007 Riga, Latvia; ingrida.mitre@rsu.lv (I.M.); dina.nitisa@rsu.lv (D.N.); valdis.pirsko@rsu.lv (V.P.)

**Keywords:** SK-BR-3, RT-qPCR, reference genes, hypoxia, gene expression, breast cancer cell line, HER2 enriched

## Abstract

Normalization of gene expression using internal controls or reference genes (RGs) has been the method of choice for standardizing the technical variations in reverse transcription quantitative polymerase chain reactions (RT-qPCR). Conventionally, *ACTB* and *GAPDH* have been used as reference genes despite evidence from literature discouraging their use. Hence, in the present study we identified and investigated novel reference genes in SK-BR-3, an HER2-enriched breast cancer cell line. Transcriptomic data of 82 HER2-E breast cancer samples from TCGA database were analyzed to identify twelve novel genes with stable expression. Additionally, thirteen RGs from the literature were analyzed. The expression variations of the candidate genes were studied over five successive passages (p) in two parallel cultures S1 and S2 and in acute and chronic hypoxia using various algorithms. Finally, the most stable RGs were selected and validated for normalization of the expression of three genes of interest (GOIs) in normoxia and hypoxia. Our results indicate that *HSP90AB1*, *DAD1*, *PFN1* and *PUM1* can be used in any combination of three (triplets) for optimizing intra- and inter-assay gene expression differences in the SK-BR-3 cell line. Additionally, we discourage the use of conventional RGs (*ACTB*, *GAPDH*, *RPL13A*, *RNA18S* and *RNA28S*) as internal controls for RT-qPCR in SK-BR-3 cell line.

## 1. Introduction

Reverse Transcription Quantitative Polymerase Chain Reaction (RT-qPCR) represents a modified variant of the popular conventional PCR with diverse applications, ranging from functional genomics to molecular medicine, virology, microbiology, and biotechnology [1]. Quantitative PCR-based assays can target both DNA (genome) and RNA (transcriptome), thereby making it an extremely powerful and important technique in molecular diagnostics [2]. While functional genomics deals with understanding the functions and interactions of genes and proteins at a genome-wide level including the role of ligands, receptors, and signaling networks that converge on transcriptional regulation [2], transcriptomic analysis, deals with ascertaining the functional significance to expression signature changes between tissues, disease states, or treatment [2]. Large-scale analysis of expression patterns is performed by RNA-Seq or high-throughput microarray analysis, however, the findings for individual genes usually are validated by RT-qPCR due to its high sensitivity, specificity, reproducibility, and broad dynamic range [2,3,4].

However, this enhanced sensitivity of RT-qPCR imposes special conditions. The protocol necessitates accurate and precise pipetting, high-quality RNA, accurate estimation of RNA concentration, and efficient reverse transcription [3]. Other considerations include standardization of RT-qPCR protocols [5], maintaining consistency of used reagents [6,7] and careful attention towards assay design, template preparation, and statistical analysis [8]. Any deviation from these requirements (by human error etc.) can introduce variations and influence the accuracy and precision of the results [9,10,11,12]. To account for such deviations, several strategies are recommended that can be incorporated in the protocol at different stages [12,13,14] ranging from ensuring that a similar sample size is chosen to using controls such as spike-in foreign RNA and reference genes (previously housekeeping genes).

Normalization using reference genes has been the method of choice by most researchers since variations in the experimental workflow will affect all genes similarly [14,15,16]. The expression of reference genes is expected to be sufficient and stable across different tissues and cell lines under varied experimental conditions [17]. However, variance in the expression of conventional reference genes like *ACTB* and *GAPDH* across different cell types has been noted [18,19,20,21,22] causing a continued search for suitable candidate genes. In addition, given the highly specific nature of RT-qPCR, the Minimum Information for Publication of Quantitative RT-PCR Experiments (MIQE) guidelines recommend using more than one reference gene for normalization unless a clear evidence of uniform expression dynamics of a single reference gene is reported for specific experimental conditions [11]. Identification and validation of novel reference genes, hence, becomes imperative for accurate normalization. In the past, reference genes have been identified using various large-scale gene expression profiling methods such as Expressed Sequence Tags (ESTs), Serial Analysis of Gene Expression (SAGE) and Microarray Analysis [23,24,25]. However, with the advent of technology, better techniques using RNA-seq data have been employed to identify stable reference genes. Several studies have previously identified novel reference genes and/or validated conventional reference genes for the study of breast cancer [26,27,28,29,30,31,32,33,34,35,36,37,38].

Breast cancer represents the most common malignant disease worldwide among women, accounting for 24% of new cancer cases and 15% of cancer-related deaths in 2018 [39] with the number predicted to almost double to 46% by 2040 [40]. With the immense burden of the disease, it becomes crucial to develop better protocols, prediction tools, diagnostics, and treatment modalities. The PAM50 (Prediction Analysis for Microarrays) represents a 50 gene classifier containing mostly hormone receptor, proliferation-related, myoepithelial and basal feature-related genes and is widely used to classify breast cancer into molecular subtypes [41,42,43]. The HER2-Enriched (HER2-E) subtype according to PAM50 is defined by higher expression of *ERBB2* along with the upregulated expression of tumor proliferation-related genes at the RNA and protein levels in comparison to other cancer types [44,45]. SK-BR-3, established in 1970 from the pleural effusion of a Caucasian female with malignant breast adenocarcinoma, is a human breast cancer cell line overexpressing *ERBB2* gene product [46]. There are contrasting views in the literature over the classification of SK-BR-3 with some authors including it in the luminal subtype [47,48], with others classifying it as HER2-E [49,50,51].

Hypoxia is one of the principal drivers of tumor progression and growth in vivo. The presence of hypoxic conditions in the cancer microenvironment is not only a recognized event in cancer development but also is sustained by the cancer cells themselves, secondary to the inflammatory processes [52,53,54]. Such a condition in the local tumor microenvironment is necessary to induce angiogenesis and release of growth factors whilst inducing structural and functional damage to the healthy surrounding tissue [54]. It is estimated that about 1.5% of the whole human genome is transcriptionally responsive to hypoxia, thereby affecting gene expression [55]. Hence, given the critical role of hypoxia in tumorigenesis and its direct impact on gene expression, we decided to investigate the stability of the chosen reference genes in different hypoxic conditions.

In the present study, we identified and validated novel reference genes that could be used to normalize qPCR data in SK-BR-3 breast cancer cell line. Additionally, we compared expression stability of newly identified genes with previously reported reference genes [26,27,28,29,30,31,32,33,34,35,36,37,38] to select suitable candidate reference genes. Our study reports for the first time to our knowledge, a comprehensive analysis, combining previous and novel candidates studied over multiple successive passages (p), in replicate cultures S1 and S2, and validated in various hypoxic conditions for SK-BR-3 cell line.

## 2. Materials and Methods

### 2.1. TCGA Transcriptomic Analysis for Selection of Novel Candidate Reference Genes

The transcriptome profiling datasets of 82 HER2-E (PAM50 classifier) breast cancer samples were downloaded from TCGA-BRCA legacy archive via R package *TCGAbiolinks* [56,57,58]. The scaled_estimate (Platform—Illumina HiSeq; file extension—rsem.genes.results) values, which represent the estimated frequency of gene/transcripts amongst the total number of transcripts that were sequenced, were obtained from the database. TPM (transcripts per million) values were generated by multiplying scaled_estimate values to a factor of 1 million (10^6^). For data analysis and visualization, the TPM values were converted to logarithmic scale using log2(TPM).

### 2.2. Gene Ontology (GO)

GO Annotation and enrichment analysis was done using a web based open-access tool—The Gene Ontology Resource, powered by Panther Classification (http://geneontology.org/ (accessed on 15 April 2021)) [59,60,61,62]. Gene ranking was based on the fold enrichment against the background frequency of total genes annotated to that term in the designated species (*Homo Sapiens*; whole genome, GO version Oct 2020, doi: 10.5281/zenodo.408174) [63]. Fisher’s Exact test with False Discovery Rate (FDR) correction was used to estimate significance with the cut-off FDR value of <0.05. The tool was further used to group genes based on functional classification.

### 2.3. Culture and Seeding Conditions

Samples were collected from the SK-BR-3 cell line (ATCC, HTB-30) that had been used in our laboratory for previous studies [64]. For consecutive passage analysis, two cultures, S1 and S2, were established from different laboratory lineages of SK-BR-3 which were cultured over five consecutive passages (p7–p11). For hypoxic exposure analysis, cultures at the level of 80–90% confluence were transferred to Xvivo System Sx2 (BioSpherix Medical; 37 °C, 5% CO_2_, 2% O_2_). The length of hypoxic exposure for acute hypoxia samples was 24 h and 72 h. To obtain chronic hypoxia samples, the cultures (*n* = 3) were fully maintained in the hypoxic environment for four consecutive passages. From each culture, 3 lysates (in triplicates) were collected per passage. Cells were cultured in RPMI-1640 (Lonza, BE12-115F), supplemented with 10% FBS (fetal bovine serum; Sigma Aldrich, F9665) at 37 °C, 5% CO_2_ with the growth medium replaced every 2–3 days. Cell passaging was performed using 1x TrypLE solution (Thermo Fisher Scientific, A12177-02). Cells were grown to 80–100% confluence in T-25 cm^2^ flasks (Sarstedt). Cell count and viability were estimated using a cell counting chamber (Improved Neubauer Hemocytometer). For further consecutive passages, cells were seeded at a density of 5000 cells/cm^2^. Three TRIzol lysates (1 × 10^6^ cells) were obtained from each passage for both cultures for RNA isolation.

### 2.4. RNA Extraction and cDNA Synthesis

Total RNA was extracted using Trizol reagent (Thermo Fisher Scientific, Waltham, MA, USA; 15596026) according to the manufacturer’s protocol. The concentration and quality of RNA were assessed by Nanodrop 2000 with the mean absorption ratios A260/280 and A260/230 checked to ensure RNA purity. RNA integrity was evaluated using 1.8% agarose gel electrophoresis. The RNA was further examined for DNA contamination by PCR for *ACTB* and *GAPDH*. The PCR reaction was performed in the presence of both positive and negative controls. No amplified PCR product was found on the agarose gel after PCR and electrophoresis of the RNA samples (except for positive controls). The cDNA synthesis reaction was carried out using the High-Capacity cDNA Reverse transcription kit (Thermo Fisher Scientific, Waltham, MA, USA; 4368814) in accordance with the manufacturer’s protocol and guidelines and was stored at −20 °C until further analysis.

### 2.5. Selection of Reference Genes and Primer Design

In total, thirteen reference genes were selected by searching for relevant literature related to various breast cancer cell lines [26,27,28,29,30,31,32,33,34,35,36,37,38]. Twelve more genes were selected from the TCGA dataset analysis and were referred to as Novel Candidate Reference genes. All selected genes are summarized in Table 1. Along with these 25 candidate reference genes, three genes of interest (target genes; GOIs) were also normalized to test the selected reference genes (Table 1). Primers for all 12 selected novel candidate reference genes, *PPIA* and *SNAI1* were designed using Primer3Plus in accordance with the MIQE guidelines [11,65] while the rest were taken from literature or from our previous study in MCF-7 cell line [66]. The gene function, primer pairs and respective melting curves of all the selected genes are presented in Supplementary File S1.

Selection of the three genes of interest was based on the data from Expression atlas (https://www.ebi.ac.uk/gxa/home (accessed on 15 April 2021)); European Bioinformatics Institute). The atlas was searched for SK-BR-3 cell line and GOIs were randomly selected based on high expression (*AURKA*; 241 TPM), medium expression (*BUB1*; 31 TPM), and low expression (*SNAI1*; 5 TPM).

### 2.6. Primer Efficiency

Standard (calibration) curves were analyzed using different concentrations and dilutions as shown in Supplementary File S1. For each reaction, 7 µL was used in a 384 well plate. Each dilution was done in triplicate for each primer pair along with appropriate non-template controls (NTC). Real-time PCR was performed using the ViiA7 RT-PCR thermocycler (Thermo Fisher Scientific). The cycling parameters were 95 °C for 10 min followed by 40 cycles of amplification at 95 °C for 15 s, 58 °C for 30 s and 72 °C for 30 s with signal acquisition. After that melting curve were obtained by signal acquisition from 58 °C to 95 °C in increments of 0.05 °C/s.

### 2.7. Reverse Transcription Quantitative PCR (RT-qPCR)

Reverse transcription quantitative PCR (RT-qPCR) was performed with 10 ng of cDNA per reaction using ViiA 7 RT-PCR thermocycler (Thermo Fisher Scientific). Triplicate reactions of each sample were done using HOT FIREPol EvaGreen qPCR Supermix (Solis Biodyne, Tartu, Estonia; 08-36-00020) on 384 well plates (Applied Biosystems™, Thermo Fisher Scientific, Waltham, MA, USA; LS4309849). The cycling parameters were 95 °C for 10 min followed by 40 cycles of amplification at 95 °C for 15 s, 58 °C for 30 s and 72 °C for 30 s. This was followed by melting curve acquisition as described above. All assays were performed with non-template controls (NTC).

### 2.8. Determination of the Least Variable Reference Genes and Validation in Hypoxic Conditions

**Scheme 1:**
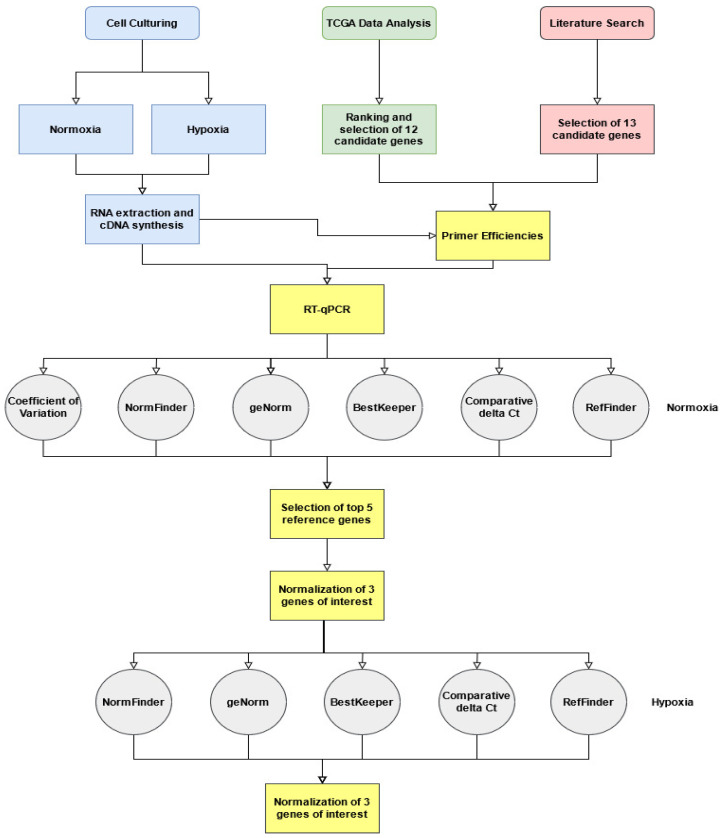
Brief overview of the workflow of analysis employed in the present study. The boxes indicate the different steps including lab wet work that was performed. The circles indicate the algorithms used for selection and identification of appropriate reference genes in SK-BR-3 breast cancer cell line. Blue boxes indicate the cellular wet lab work whilst the green and pink boxes indicate the two pipelines followed for selection of candidate reference genes. The yellow boxes indicate the major work packages and milestones in the common workflow employed. Grey circles indicate the different algorithms employed for the determination of appropriate reference genes.

The Cq values obtained from RT-qPCR were used to determine the stability of the candidate reference genes in the sequential normoxic passage samples using different algorithms (coefficient of variation—CV%, NormFinder [67], geNorm [17], BestKeeper [68], Comparative ∆Ct [69] and RefFinder [70]). The least variable reference genes were then tested in hypoxic samples to verify their stability and expression in hypoxic conditions. The working methodology and cut-off criteria for each of these algorithms is demonstrated in Supplementary File S4. Data management and storage along with descriptive analysis was done using MS Excel (Microsoft Office 365). The various algorithms mentioned above were performed using R v4.0.2 (via R studio v1.3.1056). A complete workflow employed in the present study is shown in Scheme 1.

## 3. Results

### 3.1. TCGA Analysis for Selection of Novel Reference Genes

Novel candidate reference genes were selected on the basis of HER-E breast cancer sample transcriptomic data from TCGA legacy dataset by applying the following criteria: (I) medium to high expression levels—mean (log2(TPM) ≥ 5; (II) low expression variance—standard deviation (log2(TPM)) ≤ 1; (III) no exceptional expression—no log2 (TPM) differs from the mean log2 (TPM) by a factor of two or more (criteria based on study by Li Y. et al.) [71]. Once the genes were filtered according to the criteria, CV% (Coefficient of Variation) was calculated. The lower the CV%, the more stable the expression of a candidate reference gene.

A complete list of 3363 ranked genes which fulfilled the selection criteria is available in Supplementary File S5. The candidates for validation (12 genes) were selected, based on further criteria including association with dissimilar cellular functions and that the selected genes are not subunits of the same protein as encoded by traditional reference genes. Upon consideration, from the top 10 genes, six genes were selected (*GABARAP*, *PFN1*, *UBC*, *EIF5A*, *CFL1* and *TPT1*). Three more genes were selected from ranks 11–20 (*NACA*, *DAD1* and *PSMB4*). Finally, three genes (*TUBA1B*, *RBX1* and *BSG*) were randomly selected from the top 400 ranks.

**Figure 1:**
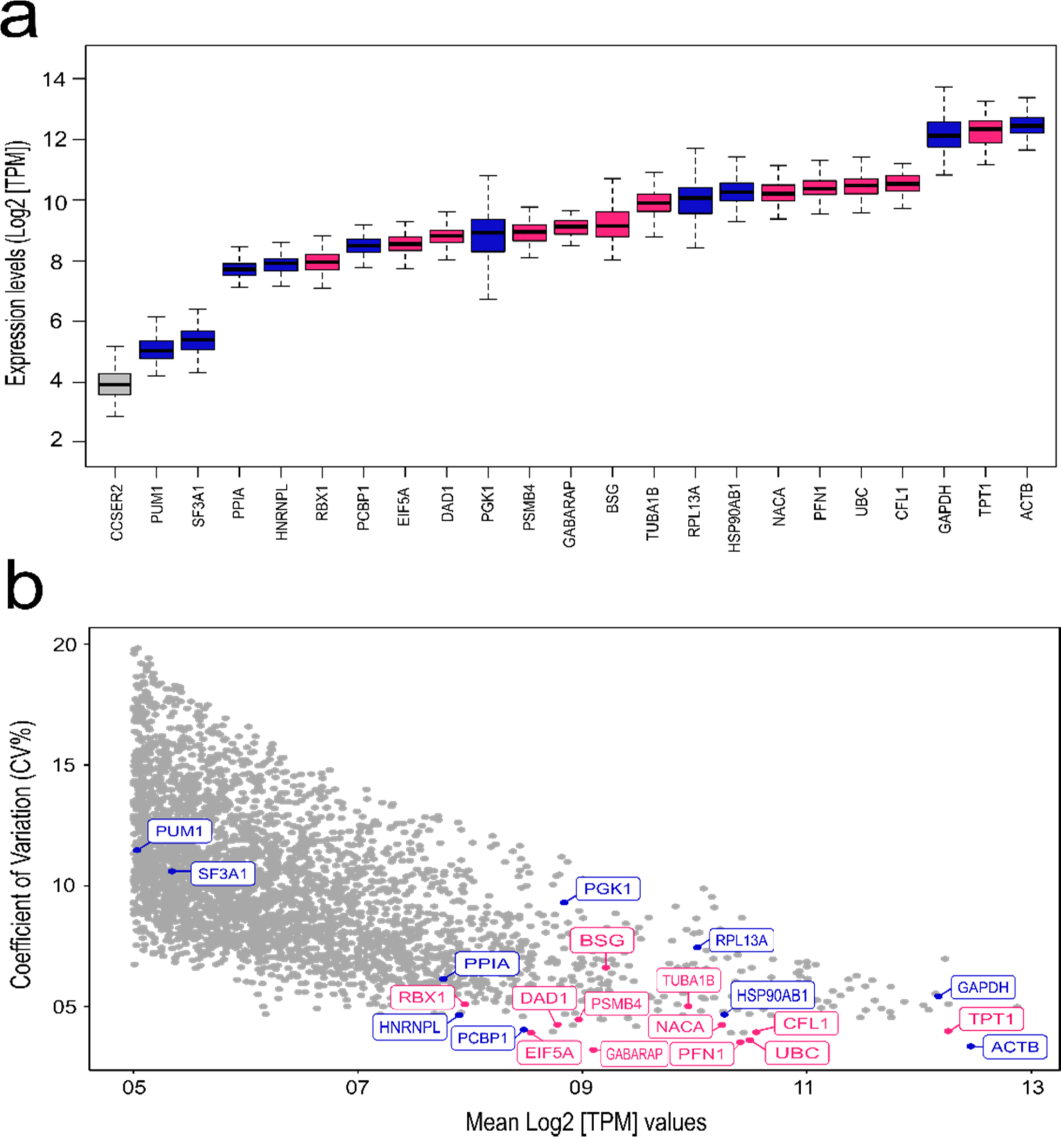
Ranking of the novel (pink) and conventional (blue) candidate reference genes. The rankings are based on TCGA database analysis. (**a**) Gene ranking based on expression levels (log2[TPM]); (**b**) Scatterplot showing the order of genes based on CV% and mean log2[TPM] values. *CCSER2* (grey boxplot) was retrieved as *FAM190B* and violated the selection criteria due to low expression levels.

Based on expression levels (measured as log2[TPM]), *ACTB*, *TPT1* and *GAPDH* showed high expression levels while *PUM1*, *SF3A1* and *PPIA* showed low expression levels in HER2-E samples (Figure 1a). Based on CV%, however, *GABARAP*, *ACTB* and *PFN1* were the genes with the least variable expression, while *PUM1*, *SF3A1* and *PGK1* were the genes with the most variable expression (Figure 1b). Genes with higher levels of expression generally were associated with lower inter-sample variation.

### 3.2. Gene Ontology (GO) Over-Representation Analysis

Based on fold enrichment (Table 2), the top ranked biological process was modulation by symbiont of the host process which included *GAPDH*, *UBC* and *PGK1*. However, the most significantly enriched biological process was cellular response to cytokine stimulus (FDR = 3.33 × 10^−03^) which included *GAPDH*, *RBX1*, *TUBA1B*, *HSP90AB1*, *RPL13A*, *UBC*, *CFL1*, *PPIA* and *PSMB4*. A complete ontology for Molecular function and Cellular Component is presented in Supplementary File S2.

### 3.3. Grouping of Genes Based on Functional Classification (Panther)

**Figure 2:**
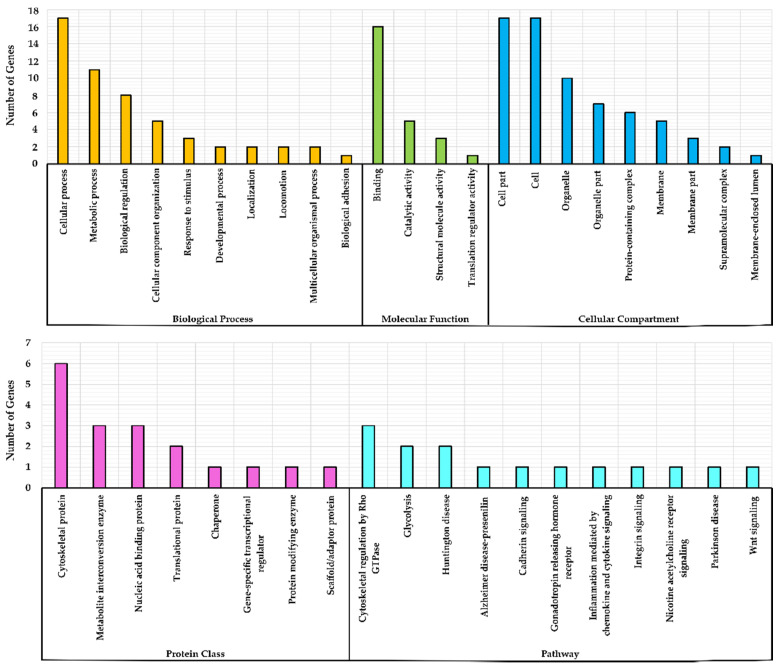
Grouping of the candidate reference genes according to Panther Functional Classification, which is further based on five different ontologies.

Panther was used to group the candidate reference genes based on function. The grouping was done across five different ontologies (Figure 2; Supplementary File S2)—GO biological process, GO molecular function, GO cellular component, protein class and pathway. Most of the genes in GO cellular component analysis were associated with cell or cell part (17 genes each) (Figure 2). In protein class classification six genes were identified as genes encoding cytoskeletal proteins (*ACTB*, *TUBA1B*, *TPT1*, *PFN1*, *CFL1* and *GABARAP*). Finally, based on pathway, three genes were associated with cytoskeletal regulation by Rho GTPase pathway (*ACTB*, *PFN1* and *CFL1*) while two genes were associated each with glycolysis (*GAPDH* and *PGK1*) and Huntington disease (*GAPDH* and *ACTB*).

### 3.4. Candidate Reference Gene Stability in SK-BR-3 Cell Line

**Figure 3:**
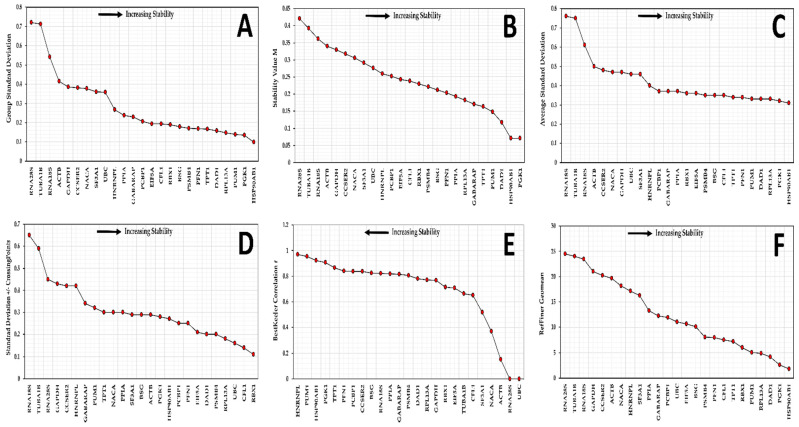
Ranking of candidate reference genes according to various algorithms (**A**) NormFinder; (**B**) geNorm; (**C**) Comparative ∆Ct; (**D**) BestKeeper; (**E**) Correlation with BestKeeper Index (BI) and (**F**) RefFinder.

In the present study, we collected three technical replicates from five consecutive passages (p7–p11) in two replicate cultures S1 and S2. The biological replicates were analyzed for all 25 candidate reference genes, thereby producing a dataset with 250 Cq values. A similar dataset was analyzed for the three genes of interest (dataset of 30 Cq values). Different algorithms were then used to analyze the stability of the reference gene expression. NormFinder, geNorm, comparative ∆Ct and RefFinder all ranked *HSP90AB1*, *PGK1*, *DAD1*, *PUM1* and *RPL13A* as the most stable genes in the consecutive passages of SK-BR-3 (Figure 3). BestKeeper, however, ranked *RBX1*, *CFL1* and *UBC* as the most stable genes. *RNA18S*, *TUBA1B* and *RNA28S* were consistently ranked as the least stable reference gene candidates by all algorithms.

### 3.5. Candidate Reference Gene Stability in Replicate Cultures

To analyze the expression stability of candidate reference genes in replicate cultures S1 and S2, we applied all algorithms separately, as shown in Supplementary File S3. Descriptive analysis revealed that *CCSER2* and *GABARAP* showed low expression levels (Cq > 28), while *RNA28S* and *RNA18S* showed high expression levels (Cq < 15). Coefficient of variation (CV%) analyses revealed that *TUBA1B* showed high variation (>50%) and hence was not a suitable candidate. Further analysis with BestKeeper revealed *ACTB*, *SF3A1*, *CFL1*, *UBC* and *NACA* to have a low/moderate correlation with the BestKeeper Index (BI). After removal of these 10 candidate reference genes, the remaining 15 genes were re-analyzed using various algorithms, and none of the remaining genes violated any criteria. Finally, cumulative rankings from both cultures revealed *HSP90AB1*, *DAD1*, *PGK1*, *RPL13A* and *PUM1* to be the top five most stable genes.

### 3.6. Selection of Reference Genes for Further Validation

Since geNorm indicated that use of two genes (Supplementary File S3) would be sufficient, we decided to select the top five least variable genes from our analysis and test different triplets (rather than in pairs, as a good practice) of the selected five genes. To select the genes, we analyzed the results obtained so far from both cultures whilst also factoring-in CV% rankings (Supplementary File S3). As a result, *HSP90AB1*, *DAD1*, *PFN1*, *RPL13A* and *PUM1* were selected for further analysis and normalization of the genes of interest.

### 3.7. Normalization of Genes of Interest (GOIs)

**Figure 4:**
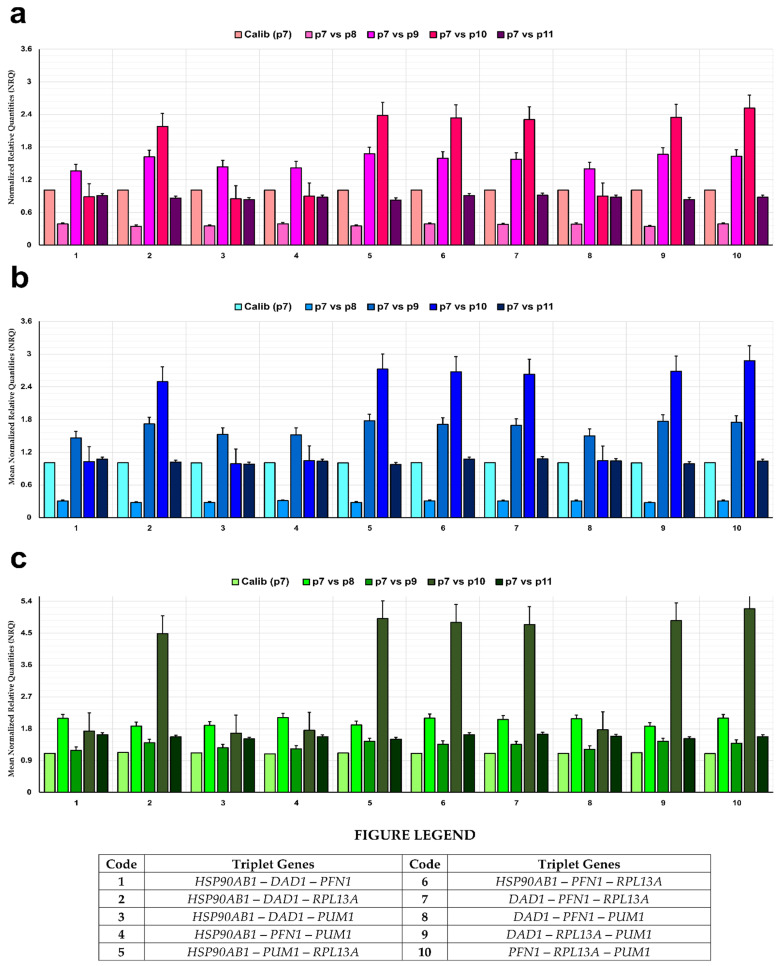
Normalization of the three genes of interest (GOIs) in sequential passage experiments of SK-BR-3 cell line (S1 + S2) for (**a**) *AURKA*; (**b**) *BUB1* and (**c**) *SNAI1*. The corresponding triplet codes shown on the x-axis are interpreted in figure legend. Error bars indicate the standard error (S.E.). Calib (p7) stands for the calibration passage 7, i.e., the initial passage which was used as an internal experimental control to obtain normalized relative quantities (NRQ) by Pfaffl’s method.

The ∆∆Ct method is the method of choice for normalization of the gene expression of genes of interest [72]. A modification of the ∆∆Ct method was employed in the present study. The modification allowed us to account for differential primer efficiencies and the use of multiple reference genes [73,74]. Subsequently, to account for primer efficiency in the equation, the efficiencies from broad and narrow range dilutions were used (Supplementary File S1). We then normalized the three genes of interest (GOIs)—*AURKA*, *BUB1* and *SNAI1*—with triplet pairs of the five chosen reference genes (10 possible triplets as shown in Figure 4). According to the guidelines, any arbitrary sample can be considered as an internal calibrator (control) for normalization without any effects on the relative quantification result. Hence, we considered passage p7 as the internal calibrator since it was the initial passage in the experiment.

For evaluation of successful normalization, the NRQs (normalized relative quantities) should be checked for patterns in expression, and the difference should be minimal between each sample after normalization [73,75]. For *AURKA*, we noticed that the expression in comparison with p7 decreased in p8 followed by increase in p9. The NRQs were found to be close to p7 levels in p10 and p11 (Figure 4a). However, this trend was not observed in the triplets with *RPL13A* (triplets 2, 5, 6, 7, 9 and 10). The expression of triplets with *RPL13A* in p9 and p10 was highly elevated in comparison to p7 (Figure 4a). Similar observations were made for *BUB1* and *SNAI1* (Figure 4b,c). This may be explained by the fact that among the five selected genes *RPL13A* had the lowest correlation r (r = 0.856) with the BestKeeper Index (Additional Table S10 in Supplementary File S3), which was also shown by NormFinder results. Hence, *RPL13A* was not an ideal candidate and we removed it from the further analysis.

### 3.8. Effects of Hypoxia on the Stability of the Selected Reference Genes

**Figure 5:**
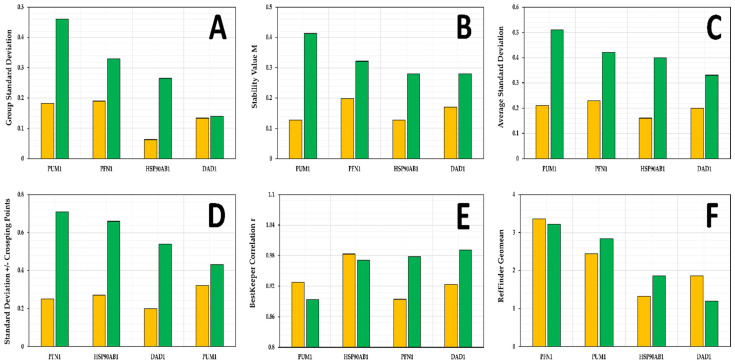
Ranking of the candidate reference genes according to various algorithms (**A**) NormFinder; (**B**) geNorm; (**C**) Comparative ∆Ct; (**D**) BestKeeper; (**E**) correlation with BestKeeper Index (BI) and (**F**) RefFinder. Yellow bars indicate the stability values for the genes in normoxia samples, while the green bars indicate the stability values for the genes in all samples (normoxia and hypoxia). The reference genes are ranked according to the stability rankings in all samples (green bars). The stability of the reference genes in the sample pool increases from left to right for all algorithms.

To evaluate the effects of hypoxia on reference gene stabilities, we applied all algorithms combining data with hypoxic sample results for the four reference genes (*HSP90AB1*, *PUM1*, *DAD1* and *PFN1*). In hypoxia, *DAD1* and *HSP90AB1* were the two most stable genes as revealed by NormFinder, geNorm, Comparative ∆Ct and RefFinder (Figure 5). BestKeeper, however, ranked *PUM1* as the most stable gene, and the *DAD1* showed the highest correlation with the BestKeeper index. Comparison of the expression stabilities of the genes in normoxia vs. hypoxia showed that the expression stability of all four genes decreased after the addition of hypoxic samples to the dataset (Figure 5). Only *DAD1* and *PFN1* displayed improved expression stability in hypoxia according to RefFinder (Figure 5F). Similarly, only these two genes improved their correlation with the BestKeeper index in hypoxia. Nonetheless, no gene violated the respective cutoffs in any of the algorithms, making them suitable reference genes.

### 3.9. Experimental Validation of Reference Genes in Hypoxic Conditions

**Figure 6:**
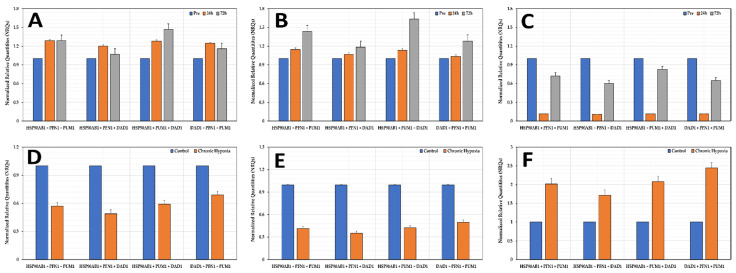
Normalization (Pfaffl’s method) of the three genes of interest (GOIs) in SK-BR-3 cell line in acute hypoxia (**A**–**C**) and chronic hypoxia (**D**–**F**) for (**A**,**D**) *AURKA*; (**B**,**E**) *BUB1* and (**C**,**F**) *SNAI1*.

We found that after normalization *AURKA* was slightly upregulated (Figure 6A) after 24 h of hypoxic exposure while after 72 h, different triplets indicated different results. The normalization factor (NF) was within the acceptable limits (NF < 2 to 3-fold relative to the average), thus eliminating potential causative issues, e.g., starting material quantity or quality, or a problem with one of the reference genes (either not stably expressed, or not adequately measured) [75]. As pointed out by the other authors [34,75], the choice of calibrator sample (reference genes) does not influence the relative quantification result.

Although numbers may differ, the actual fold differences between the samples remain identical, therefore the results are fully equivalent and only rescaled. The expression of *BUB1* was sequentially increasing, while *SNAI1* after an initial (24 h) decrease became upregulated, but still below the control levels (Figure 6). Both *AURKA*, and *BUB1* were downregulated (−0.78 and −1.26 average log2 fold change, respectively) in chronic hypoxia, when normalized by all four reference gene triplets (Figure 6). However, *SNAI1* was upregulated in chronic hypoxia (+1.03 average log2 fold change). Based on our analysis, we can conclude that the selected reference gene triplets were able to successfully normalize the genes of interest irrespective of the expression levels of gene of interest in SK-BR-3 cell line (*AURKA*—high expression; *BUB1*—medium expression; *SNAI1*—low expression).

## 4. Discussion

A pivotal aspect in any gene expression study is the selection of appropriate internal controls that are expressed uniformly irrespective of culturing conditions, experimental treatment, nutrient stress etc. These controls (or references) normalize any variations in starting quantities, calibration issues or poor pipetting, thereby providing accurate results. However, complex gene-gene interactions and environmental effects on gene expression complicate the identification of such controls. Another layer of complexity in this pursuit is added by intrinsic heterogeneity of the cancer cells. Several tools and algorithms (NormFinder, geNorm, BestKeeper, Comparative ∆Ct and RefFinder) have been developed that can aid in sorting, selection, and validation of the reference genes. However, the considerations regarding the applicability of these algorithms and the acceptable cut-off limits are often misinterpreted or misapplied. Finally, the identification and extensive validation of the reference genes for each type of biological object in different conditions may not be feasible in every instance. Such studies are often time and resource (labor, financial) consuming. In the present study, we have identified and validated novel reference genes for normalization of RT-qPCR results in SK-BR-3 breast cancer cell line.

In the past the TCGA database has been commonly used to identify novel and reliable candidate genes in different cancer types [37,38], but it has never been investigated individually for HER2-E subtype of breast cancer. Our analysis of TCGA legacy data revealed that *GABARAP*, *ACTB* and *PFN1* were the top three genes with the least variation amongst the samples (Figure 1b). However, upon validation in the SK-BR-3 cell line, we found that the least variation amongst the samples was displayed by *RBX1*, *UBC* and *CFL1* (Supplementary File S3). These differences between TCGA database and RT-qPCR results are normal and expected. Firstly, the database represents a heterogenous mixture of similar subtypes while the SK-BR-3 is a single cell line, and therefore constitutes a more homogenous sample. Secondly, all samples of TCGA repository come from primary untreated tumors collected from different institutes. Thirdly, there is an inherent possibility of bias in the biorepository generation process, stemming from different institutional research interests, operative protocols, or patient populations [76]. Additionally, it must be considered that tissue specimens (TCGA data) in addition to cancer and normal mammary cells contain various types of stromal and immune cells, which are absent from the cell culture samples. This type of heterogeneity also may have effects on the overall transcription levels and stability. The database accommodates for most of the possible gene expression variations in tissues (in vivo) and, since cell lines like SK-BR-3 have been under constant cultivation over long-term, they tend to behave like outliers when compared to the TCGA database. Nonetheless, such extrapolation, bridging and application of in vivo data to in vitro conditions enable us to find common core genes which are uniformly expressed in both scenarios.

Gene Ontology (GO) analysis (Table 2) revealed that the candidate genes selected based on the TCGA samples and literature were most significantly enriched in cellular response to cytokine stimulus (GO:0071345; *CFL1*, *GAPDH*, *HSP90AB1*, *PPIA*, *PSMB4*, *RBX1*, *RPL13A*, *TUBA1B* and *UBC*). Indeed, it is well known that breast cancer cells respond to various cytokines in their microenvironment. The cells have been shown to evade cytokines such as TGF-β in the early stages (due to its anti-proliferative effects), however, in the later stages TGF-β stimulates the progression of the disease by inducing epithelial to mesenchymal transition (EMT type 3) [77,78,79]. IL-1 (adipokine) is known to increase the aromatase activity in SK-BR-3 cells, resulting in the generation of bioactive estrogens and increased cellular proliferation [80]. Other cytokines like IL-6, IL-19, IL-20, TGF-α, TNF-α, and IL-23 are also known to promote cancer progression [77]. Using data available from Mouse Genome Informatics (MGI; http://www.informatics.jax.org/ (accessed on 12 June 2021)), *CFL1* was associated with cellular response to IL-1, IL-6 and TNF, *GAPDH* and *RPL13A* were associated with response to IFN-γ, while *HSP90AB1* and *TUBA1B* were associated with the response to IL-4. Apart from the cytokine response, the genes were found to be enriched in viral life cycle (GO:0019058; *BSG*, *HSP90AB1*, *PCBP1*, *PPIA* and *UBC*) and positive regulation of viral processes (GO:0048524; *BSG*, *PFN1* and *PPIA*). Evidence of various virus-related DNAs like EBV (Epstein-Barr virus), HPV (Human Papillomavirus), BLV (bovine leukemia virus) and MMTV (Mouse mammary tumor virus) has been found in breast cancers [81,82,83,84]. In fact, Lawson et al., demonstrated the presence of HPV-associated pre-malignant koilocytes in normal and malignant breast tissues [85], indicating possible oncogenic correlation between the viruses and breast cancer. Using data from MGI, we found that *BSG* was associated with positive regulation of viral entry into the host cell, while *HSP90AB1* was associated with virion attachment to the host cell. On the other hand, *PFN1* and *PPIA* were found to be associated with positive regulation of viral transcription and viral genome replication, respectively.

To understand the role of the four validated reference genes in metabolic processes, GO biological processes analysis in association with data from MGI (http://www.informatics.jax.org/ (accessed on 12 June 2021)) were investigated. The genes were found to be associated with negative regulation of apoptosis (*DAD1*) and nucleobase containing compound metabolic process (Table 2). *HSP90AB1* was found to be associated with positive regulation of activities of telomerase, phosphoprotein phosphatase and protein serine/threonine kinase. *PFN1* was found to play a role in the positive regulation of DNA metabolic processes and of transcription by RNA polymerase II, while *PUM1* was associated with the regulation of mRNA stability and the production of miRNAs (microRNAs). Based on Molecular function, *HSP90AB1* and *PFN1* shared two common ontologies—RNA binding and Cytoskeletal protein binding (Supplementary File S2), both of which seemed to be downregulated in hypoxic conditions (for *PFN1*, fold change was 0.68 and 0.45 after 24 and 72 h of acute hypoxia, respectively, and 0.89 in chronic hypoxia). With respect to cellular component analysis the regulation of different genes (even from the same ontology) during hypoxia was divergent/unrelated. Whilst *PUM1*, *HSP90AB1* and *PFN1* shared two ontologies, nuclear and cytosol-associated genes, the genes were differently regulated with *PUM1* showing upregulation while the other two showed downregulation in gene expression.

Our analysis revealed that the four reference genes (*HSP90AB1*, *DAD1*, *PUM1* and *PFN1*), when used in any combination of three, successfully normalized the genes of interest (*AURKA*, *BUB1* and *SNAI1*) in both hypoxic and normoxic conditions over multiple passages. *HSP90AB1*, previously known as *HSPCB*, was first described as a candidate reference gene by Jacob et al. [31] in a variety of 25 different cancer cell lines. However, the only breast cancer line in that study was MCF-7 (Luminal A subtype). The gene has been included among the most stable reference genes in other tissues/organs, e.g., ovary, muscle tissue, adipose tissue etc. [86,87]. Heat Shock Proteins like *HSP90AB1* have been previously shown to be downregulated in response to hypoxia in pig adipose-derived stromal/stem cells [88] as well as in human hepatocytes [89]. Such downregulation (fold expression change of 0.71 after 24 h and 0.88 after 72 h in acute hypoxia and 0.52 in chronic hypoxia) appeared to have an impact on the stability of the gene by marginally lowering the expression stability in hypoxic conditions (Figure 5).

*DAD1*, a novel reference gene identified in the present study, is a small integral membrane protein of the oligosaccharyltransferase (OST) enzyme complex involved in the highly conserved asparagine-linked glycosylation of proteins in all eukaryotic cells [90]. The gene has been shown to be involved in apoptosis. Loss of *DAD1* gene led to apoptosis in hamster cell lines and yeast cells [91,92]. In fact, *DAD1* was preferentially expressed in hepatocellular carcinoma (HCC) and prostate cancer cells [93,94]. Hence, it has been postulated that high expression of *DAD1* in HCC cells can activate OST and block apoptosis, thereby enhancing tumor cell survival [93]. We speculate that a similar role of the gene in the SK-BR-3 breast cancer cell line could explain its consistent and stable expression. In A431 epithelial carcinoma cells, *DAD1* was upregulated in hypoxic conditions (by a fold change of 1.2) after exposure of 72 h [95]. In our results, however, *DAD1* was downregulated by 0.64-fold change after exposure to acute hypoxia for 24 and 72 h. The expression then increased and approached normoxia levels during chronic hypoxia (fold change 0.95). The differences in our results from those in A431 cells could be explained by the cardinal differences in background transcriptome and epigenome of A4321 and SK-BR-3 cells. Furthermore, there were differences in culturing conditions. While our cells were exposed to 2% O_2_ in acute hypoxia, the A431 cells were exposed to <0.1% O_2_ [95]. Our results suggest that SK-BR-3 cells after an initial shock phase quickly adapted to chronic hypoxic conditions and continued to express core genes in levels similar to normoxia. This is also supported by the fact that the expression stability of *DAD1* improved in hypoxia, i.e., it was expressed even more stably after exposure to hypoxia.

*PFN1*, another novel reference gene identified in the present study, is often regarded as the founding member of its family constituting four profilin genes (*PFN1*, *PFN2*, *PFN3* and *PFN4*). These genes have been associated with almost every aspect of cellular functions including proliferation, survival, motility, endocytosis, membrane trafficking, mRNA splicing as well as gene transcription [96,97]. The overexpression of *PFN1* could negatively regulate cancer cell motility in breast cancer cells [98]. Additionally, it has been demonstrated that in triple negative breast cancer cell lines, overexpression of *PFN1* suppresses AKT (serine-threonine kinase) activation via upregulation of *PTEN* (phosphatase and tensin homolog) [99], indicating the tumor-suppressive character of *PFN1* gene. Similar observations have also been reported in pancreatic cancer cells [100]. These findings are of interest since the expression of *PFN1* in the SK-BR-3 cell line seems to be uniform and stable despite its anti-tumorigenic nature. In fact, depletion of *PFN1* in breast cancer cells has enabled hyper-migratory phenotype in vitro and enhanced hematogenous dissemination from primary tumor in vivo [101]. We can hypothesize that some downstream regulation is at play, which leads to decreased *PFN1* protein levels in breast cancer cells despite rather uniform expression of the gene.

Finally, the fourth reference gene identified for the SK-BR-3 cell line, *PUM1* has been previously described as a candidate reference gene in various cancers including breast cancers [28,33,35]. The gene has been associated with cancer cell growth, migration, and invasion [102]. In fact, amongst the four selected reference genes in our study, only *PUM1* showed an increase in expression fold change in hypoxia. The fold change was 0.80 and 1.13 after 24 h and 72 h of acute hypoxia, respectively, and reached 1.56 in chronic hypoxia. These findings suggest that *PUM1* may be more involved in the regulation of cellular functions under hypoxia in comparison to the other identified reference genes. Another candidate gene, *RPL13A*, has been previously described as a stable reference gene in breast cancers [27,33]. The expression of *RPL13A* was quite stable in our analysis, however, it did not yield successful normalization of the genes of interest.

Various other reference genes in the past have been described to be suitable reference genes in breast cancers including *GAPDH*, *ACTB*, *PPIA* and *RNA28S/RNA18S* [26,29,32,34]. However, there is also contrary evidence suggesting against the use of these genes as references [18,19,20,21,22]. The use of *GAPDH* as a reference gene has long been a topic of debate. It is overexpressed in cervical, prostate, pancreatic and lung cancers, and it has been the least stable gene in multiple studies [29,34,103,104]. Similar concerns have been raised concerning use of *ACTB* as a single internal control [28]. *RNA18S* and *RNA28S* have been reported previously to be stable reference gene candidates [34], however, concerns regarding the absence of purified mRNA samples and their relatively high abundance compared to the target mRNAs have been reported [17]. Both *RNA28S* and *RNA18S* were highly expressed (mean Cq = 7–8) in the present study and in our study of MCF-7 cell line [66]. Secondly, these two genes are not included in the TCGA database, which makes it difficult to compare their expression between in vivo and in vitro scenarios.

*PPIA* along with *ACTB* was found to be the most stable reference gene for basal type breast cancer cell lines in hypoxic and serum deprived conditions [36]. However, our analysis revealed that the expression of *PPIA* was moderately stable and ranked ninth in the combined analysis (Supplementary File S3). In our study of MCF-7 breast cancer cell line, we identified *GAPDH-CCSER2-PCBP1* triplet as the most stable reference gene triplet which could be used to normalize the expression of genes of interest in various nutrient stress conditions [66]. However, the expression of *CCSER2* was extremely variable in the present study. The gene did not reach the thresholds set for TCGA analysis, and the expression of the gene was low (mean Cq = 27.4). Hence, we eliminated it from our analysis in early stages. *PCBP1* and *GAPDH* were among the top 15 most stable reference genes in our analysis (Supplementary File S3). Interestingly, *GABARAP* was identified to have the least variation (CV%) among our novel candidate reference genes, however, the gene was associated with low expression and poor primer efficiency when confirmed by RT-qPCR (Supplementary Files S1 and S3).

Nonetheless, the results of the present study are constrained by some limitations. First, the expression stabilities of the reference genes were validated in vitro only. Second, these reference genes were tested in normoxic and hypoxic conditions only. Their use for normalization of expression in other conditions such as nutrient stress, drug treatment etc. remains to be validated. Finally, given the heterogeneous behavior of cancer cells, there is a need for inter-laboratory validation to further confirm our results. The major question that arises is how many more reference genes can we identify. Although a precise number will be difficult to predict, the estimates in normal human tissues (using ESTs) predict the numbers to be in the range of 3100 to 6900 genes [23], thereby making a plethora of reference genes still waiting to be identified and validated that could be more promising than the ones previously reported. However, we agree with other authors that rather than testing thousands of genes, we need to validate a panel of reference genes whose expression under varying conditions can be proven to be as minimally variable and as robust as possible [35]. Accordingly, based on our analysis, we suggest the use of *HSP90AB1*, *DAD1*, *PFN1* and *PUM1* in any combination of three (triplet), thereby giving other researchers not one but four different combinations to choose from based on their individual experimental designs and needs.

## 5. Conclusions

Based on the results of the present study, we suggest the use of *HSP90AB1*, *DAD1*, *PFN1* and *PUM1* in any combination of threes (triplet) for normalization of the expression of genes of interest in SK-BR-3 breast cancer cell line. The conventional RT-qPCR reference genes such as *ACTB*, *GAPDH*, *RPL13A*, *RNA18S*, *RNA28S*, as well as *CCSER2* and *GABARAP* are not appropriate for use as reference genes in the SK-BR-3 cell line.

## Figures and Tables

**Table 1:** Summary table of candidate reference genes and genes of interest (GOI).

Cell Line	Source	Selected Candidate Reference Genes
Breast cancer various cell lines	Literature	*ACTB* [26,29,32,34], *CCSER2* [35], *GAPDH* [34], *HNRNPL* [37], *HSP90AB1* [31], *PCBP1* [37], *PGK1* [30], *PPIA* [36], *PUM1* [28,33,35], *RNA18S* [26,34], *RNA28S* [66], *RPL13A* [27,33], *SF3A1* [32,38]
HER2-E tissue samples	TCGA (Novel)	*BSG*, *CFL1*, *DAD1*, *EIF5A*, *GABARAP*, *NACA*, *PFN1*, *PSMB4*, *RBX1*, *TPT1*, *TUBA1B*, *UBC*
Genes of Interest (GOI)	Expression Atlas	*AURKA*, *BUB1*, *SNAI1*

**Table 2:** Gene Ontology (Biological Process) of candidate reference genes ranked by fold enrichment.

GO ID	GO Term	No. of Genes *	Fold Enrichment	Raw *p* Value	FDR
GO:0044003	Modulation by symbiont of host process	3	46.10	4.24 × 10^−05^	3.05 × 10^−02^
GO:0006090	Pyruvate metabolic process	3	38.31	7.20 × 10^−05^	3.68 × 10^−02^
GO:0061418	Regulation of transcription from RNA polymerase II promoter in response to hypoxia	3	34.87	9.43 × 10^−05^	4.27 × 10^−02^
GO:0048524	Positive regulation of viral process	3	32.67	7.14 × 10^−06^	1.03 × 10^−02^
GO:0019058	Viral life cycle	5	20.33	4.26 × 10^−06^	7.52 × 10^−03^
GO:0006417	Regulation of translation	5	12.12	4.90 × 10^−05^	3.26 × 10^−02^
GO:0071345	Cellular response to cytokine stimulus	9	7.86	8.38 × 10^−07^	3.33 × 10^−03^
GO:0043066	Negative regulation of apoptotic process	7	6.96	4.13 × 10^−05^	3.12 × 10^−02^
GO:0006139	Nucleobase containing compound metabolic process	11	3.64	6.05 × 10^−05^	3.56 × 10^−02^
GO:0010604	Positive regulation of macromolecule metabolic process	12	3.05	1.33 × 10^−04^	5.02 × 10^−02^
GO:0043170	Macromolecule metabolic process	17	2.43	2.16 × 10^−05^	2.01 × 10^−02^

* No. of genes indicates the number of genes from the input selected candidate reference genes that are represented by the respective GO term.

## Data Availability

The full TCGA dataset (after processing and ranking for HER2-E subtype) is available as Supplementary File S5. The RT-qPCR raw datasets and metadata analyzed are available from corresponding authors upon reasonable request.

## Supplementary Materials

The following are available online at www.mdpi.com/10.3390/genes12101631/s1, Supplementary File S1: Primer Description and Efficiency, Supplementary File S2: Gene Ontology, Supplementary File S3: Replicate Culture Analysis, Supplementary File S4: Description of Algorithms, and Supplementary File S5: TCGA filtered ranking for HER2-E samples.
