# Supplementary material for: Identification of Novel Endogenous Controls for qPCR Normalization in SK-BR-3 Breast Cancer Cell Line
Source: Genes (Basel). 2021 Oct 17;12(10):1631. doi: 10.3390/genes12101631 (PMC8535678; doi:10.3390/genes12101631)
Supplement: Supplementary file 1 [file genes-12-01631-s001.zip › Supplementary Files/Jain et al. Appendix 1.pdf]

# APPENDIX - 1
# DESCRIPTION OF ALGORITHMS

Identification of novel endogenous controls for qPCR normalization in SK-BR-3 breast cancer cell line

Authors: Nityanand Jain*, Ingrida Mitre, Dina Nitisa, Valdis Pirsko and Inese Cakstina*

*** For Correspondence:**

Laboratory of Molecular Genetics
Institute of Oncology
Riga Stradins University
16 Dzirciema street
Riga
Latvia (LV-1007)

**Email:** nityapkl@gmail.com (NJ); inese.cakstina@rsu.lv (IC)

**Additional Table 1.** Description and criteria for different algorithms/approaches used in the present study to determine most stable reference gene pair

| Algorithm/ Approach | Criteria | Notes | Reference |
|---|---|---|---|
| Coefficient of Variance (CV%) | Homogenous sample CV < 25% and Heterogenous sample CV < 50% | Also known as Relative Standard Deviation, is calculated as the ratio of the standard deviation and mean of linear Cq values ($2^{-Cq}$). A lower CV% indicates higher gene expression stability and visa versa. | 1,2,3 |
| NormFinder | Lower the group standard deviation, higher the expression stability of the gene | Estimates the overall variation in gene expression for each gene and delivers a stability value that identifies not only the most stable gene but also the best control gene. Stability of gene is presented as an estimate of combined intra- and inter-group variation of individual gene. | 4 |
| geNorm | Lower the M value, more stable the gene;<br>M < 1 for heterogenous tissue like breast cancer | Calculates M value for each candidate gene based on pairwise comparisons. Uses stepwise exclusion method of the least stable genes by calculating average M values. | 5 |
| | Vn/Vn+1 < 0.15 | geNorm can also estimate optimal number of genes required for accurate normalization of expression data. Vn represents the number of genes suitable for normalization. However, in case Vn = 2, it is recommended to consider three stable internal controls. | |
| BestKeeper | Any of three criteria:<br>1) S.D ± C.P ≤ 1<br>2) S.D. ± x-fold ≤ 2<br>3) High coefficient of correlation r | Analyzes the expression stability of reference genes using crossing points (C.P) to decide whether the genes are differentially expressed under the applied conditions or not. | 6 |

| | | | |
|---|---|---|---|
| Comparative ΔCt | Lower the standard deviation, higher the expression stability of the gene | Compares the relative expression of pairs of candidate reference genes within each sample to identify and rank the most stable genes. For a stable gene, it should be strongly expressed, displays minimum fluctuations and is independent of expression of other genes.<br><br>Further, if ΔCq values of two genes fluctuate when analyzed in different samples, it can be concluded that one or both genes are variably expressed. Conversely, if the ΔCq values remain constant, then both genes are either expressed stably or are co-regulated among the samples. | 7 |
| RefFinder | Lower the geometric mean, the higher the expression stability of the gene | Measures geometric mean of attributed weights by NormFinder, geNorm, BestKeeper and Comparative ΔCt to generate an overall final ranking. | 8 |

## References


1. Venkat K.S, Nirmal K.S., Charbel M., Julien G. Optimal Use of statistical methods to validate reference gene stability in longitudinal studies. Plos One. 2019. 14(7): e0219440.
2. Jan Hellemans, Geert Mortier, Anne De Paepe, Frank Speleman, Jo Vandesompele. qBase relative quantification framework and software for management and automated analysis of real-time quantitative PCR data. Genome Biology. 2007 Feb. 8(2): R19.
3. D'haene B., Hellemans J. The importance of quality control during qPCR data analyis. Int. Drug Discov. 2010; 18–24.
4. Andersen CL, Jensen JL, Orntoft TF. Normalization of real-time quantitative reverse transcription-PCR data: a model-based variance estimation approach to identify genes suited for normalization, applied to bladder and colon cancer data sets. Cancer research. 2004. 64:5245-5250.
5. Vandesompele J, De Preter K, Pattyn F, Poppe B, Van Roy N, et al. Accurate normalization of real time quantitative RT-PCR data by geometric averaging of multiple internal control genes. Genome Biol 3: Research0034. 2002.
6. Pfaffl MW, Tichopad A, Prgomet C, Neuvians TP. Determination of stable housekeeping genes, differentially regulated target genes and sample integrity: BestKeeper--Excel-based tool using pair-wise correlations. Biotechnology letters. 2004. 26:509-515.
7. Silver N, Best S, Jiang J, Thein SL. Selection of housekeeping genes for gene expression studies in human reticulocytes using real-time PCR. BMC molecular biology. 2006. 7:33.
8. F Xie, P Xiao, D Chen, L Xu, B Zhang. miRDeepFinder: a miRNA analysis tool for deep sequencing of plant small RNAs. Plant molecular biology. 2012. 80 (1), 75-84.
