# Supplementary material for: Identification of Novel Endogenous Controls for qPCR Normalization in SK-BR-3 Breast Cancer Cell Line
Source: Genes (Basel). 2021 Oct 17;12(10):1631. doi: 10.3390/genes12101631 (PMC8535678; doi:10.3390/genes12101631)
Supplement: Supplementary file 1 [file genes-12-01631-s001.zip › Supplementary Files/Jain et al. Supplementary File S1.pdf]

SUPPLEMENTARY FILE S1
PRIMER DESCRIPTION & EFFICIENCY

Identification of novel endogenous controls for qPCR normalization in SK-BR-3 breast cancer cell line

Authors: Nityanand Jain*, Ingrida Mitre, Dina Nitisa, Valdis Pirsko and Inese Cakstina*

As Adapted and Modified from our Previous Study:

Jain N, et al. Selecting suitable reference genes for qPCR normalization: a comprehensive analysis in MCF-7 breast cancer cell line. BMC Mol Cell Biol. 2020 Sep 25;21(1):68. doi: 10.1186/s12860-020-00313-x. PMID: 32977762; PMCID: PMC7519550.

*** For Correspondence:**

Laboratory of Molecular Genetics
Institute of Oncology
Riga Stradins University
16 Dzirciema street
Riga
Latvia (LV-1007)

Email: nityapkl@gmail.com (NJ); inese.cakstina@rsu.lv (IC)

INDEX

1) Additional Table 1 – Description of selected reference genes and genes of interest
2) Additional Table 2 – Primer sequences of selected reference genes and genes of interest
3) Additional Table 3 – Primer efficiencies using Calibration (Standard) curve
4) Additional Figures 1-4 – Melting curves of reference genes and genes of interest
5) References

Additional Table 1. Description of the selected candidate reference genes and genes of interest

Gene Symbol	Gene Name	Molecular Function	Accession Number	Chromosomal Localisation
ACTB	β – Actin	Cytoskeleton (Contractile apparatus)	NM_001101	7p22 – p12
GAPDH	Glyceraldehyde 3-phosphate dehydrogenase	Glycolytic enzyme	NM_002046	12p13.31
RPL13A	Ribosomal protein L13a	Ribosome subunit, translation	NM_012423	19q13.33
PGK1	Phosphoglycerate kinase 1	Glycolytic enzyme	NM_000291	Xp21.1
HSP90AB1	Heat shock protein 90kDa beta	Signal transduction, Protein folding	NM_007355	6p21.1
RNA28S	28S ribosomal RNA	Ribosome subunit, translation	NR_003287	Unknown
RNA18S	18S ribosomal RNA	Ribosome subunit, translation	NR_003286	Unknown
PUM1	Pumilio RNA binding family member 1	RNA binding protein encoding	NM_001020658	1p35.2
CCSER2	Coiled coil serine rich protein 2	Microtubule binding protein encoding	NM_018999	10q23.1
HNRNPL	Heterogeneous Nuclear Ribonucleoprotein L	Formation, processing & packaging of mRNA	NM_001005335	19q13.2
PCBP1	Poly (rC) binding protein 1	RNA binding protein encoding	NM_006196	2p13.3
SF3A1	Splicing factor 3a subunit 1	Spliceosome assembly & pre-mRNA splicing	NM_005877	22q12.2
DAD1	Defender against cell death 1	Core component protein of multi-subunit oligosaccharyl-transferase	NM_001344	14q11.2
PSMB4	Proteasome 20S subunit beta 4	Proteolytic degradation of intracellular proteins	NM_002796	1q21.3
BSG	Basigin (Ok blood group)	Targets monocarboxylate transporters to plasma membrane	NM_001728	19p13.3
TUBA1B	Tubulin alpha 1b	Microtubule protein	NM_006082	12q13.12
RBX1	Ring-box 1	Ubiquitin-protein transferase activity	NM_014248	22q13.2

CFL1	Cofilin 1	Regulates actin cytoskeleton dynamics, mitosis, cytokinesis	NM_005507	11q13.1
UBC	Ubiquitin C	Ubiquitin protein ligase binding	NM_021009	12q24.3
PFN1	Profilin 1	Actin binding, cytoskeletal functions	NM_005022	17p13.2
EIF5A	Eukaryotic translation initiation factor 5A	mRNA binding protein, translation elongation	NM_001970	17p13.1
TPT1	Tumor protein, translationally-controlled 1	Calcium binding, microtubule stabilization	NM_001286272	13q14.13
NACA	Nascent polypeptide associated complex subunit alpha	Cardiac and muscle (myotubes) specific transcription factor	NM_005594	12q23-q24.1
PPIA	Peptidylprolyl isomerase A	Accelerates protein folding	NM_021130	7p13
GABARAP	GABA type A receptor associated protein	Ubiquitin like modifier, apoptosis, autophagy	NM_007278	17p13.1
AURKA*	Aurora Kinase A	Mitotic centrosomal protein kinase (controls chromosome segregation during mitosis)	NM_003600	20q13.2
BUB1*	BUB1 mitotic checkpoint serine/ threonine kinase	Spindle assembly checkpoint signaling; chromosome alignment	NM_004336	2q13
SNAI1*	Snail family transcriptional repressor 1	Induction of epithelial to mesenchymal transition (EMT)	NM_005985	20q13.13

*Genes that were used as gene of interest for normalization by candidate reference genes.

Additional Table 2. Primers for the selected candidate genes and genes of interest

Gene Symbol	Primer Pair (F - Forward; R – Reverse)	Amplicon Length (bp)	Annealing Temperature (⁰C)	Primer Reference
ACTB	F: 5`- CACCATTGGCAATGAGCGGTTC - 3′<br>R: 5`- AGGTCTTTGCGGATGTCCACGT – 3`	135	58	[1]
GAPDH	F: 5`- GACAGTCAGCCGCATCTTCT - 3`<br>R: 5`- TTAAAAGCAGCCCTGGTGAC - 3`	127	58	[1]
RPL13A	F: 5`- CTATGACCAATAGGAAGAGCAACC - 3`<br>R: 5`- GCAGAGTATATGACCAGGTGGAA – 3`	121	58	[2]
PGK1	F: 5`- CTTAAGGTGCTCAACAACATGG - 3`<br>R: 5`- ACAGGCAAGGTAATCTTCACAC - 3`	119	58	[3]
HSP90AB1	F: 5`- CTCTGTCAGAGTATGTTTCTCGC - 3`<br>R: 5`- GTTTCCGCACTCGCTCCACAAA - 3`	114	58	[1]
RNA28S	F: 5`- CAGGGGAATCCGACTGTTTA - 3`<br>R: 5`- ATGACGAGGCATTTGGCTAC - 3`	174	58	[3]
RNA18S	F: 5`- CGGCTACCACATCCAAGGAA - 3`<br>R: 5`- GCTGGAATTACCGCGGCT – 3`	187	58	[4]
PUM1	F: 5`- AGTGGGGGACTAGGCGTTAG - 3`<br>R: 5`- GTTTTCATCACTGTCTGCATCC -3`	111	58	[5]
CCSER2	F: 5`- GACAGGAGCATTACCACCTCAG - 3`<br>R: 5`- CTTCTGAGCCTGGAAAAAGGGC – 3`	143	58	[6]
HNRNPL	F: 5`- ACAAACCCCAATCTCAGTGG - 3`<br>R: 5`- CCCTCATCATGGTAATGGCT – 3`	140	58	[7]

PCBP1	F: 5`- TGATCATCGACAAGCTGGAG - 3`<br>R: 5`- TCTTTGATCTTACACCCGCC – 3`	145	58	[7]
SF3A1	F: 5`- AAGGGTCCAGTGTCCATCAAAGT – 3`<br>R: 5`- GCCATGTTGTAGTAAGCCAGTGAG – 3`	224	58	[8]
DAD1	F: 5` - CAGTTCGGTTACTGTCTCCTCG - 3`<br>R: 5` - GGAGATGCCTTGGAAATCCGCT - 3`	147	58	Present Study
PSMB4	F: 5` - CGTCCACTCCCGATTCCTTC - 3`<br>R: 5` - AATGCGAGAGATGTTGCGGA- 3`	188	58	Present Study
BSG	F: 5` - GGCTGTGAAGTCGTCAGAACAC - 3`<br>R: 5` - ACCTGCTCTCGGAGCCGTTCA - 3`	149	58	Present Study
TUBA1B	F: 5` - TGGAGGAAGGCGAGTTTTCA - 3`<br>R: 5` - GCAGGGCCAAAAGGAATGGAT - 3`	142	58	Present Study
RBX1	F: 5` - CCTCTGGGCCTGGGATATTG - 3`<br>R: 5` - TACAGACTCCCCATGCGACA - 3`	134	58	Present Study
CFL1	F: 5` - GCAACCTATGAGACCAAGGAGAG – 3`<br>R: 5` - TCTTGATGGCGTCCTTGGAGCT – 3`	118	58	Present Study
UBC	F: 5` - ACTCTGCACTTGGTCCTGC - 3`<br>R: 5` - GAATGCAACAACTTTATTGAAAGGA - 3`	103	58	Present Study
PFN1	F: 5` - ACGTTCGTCAACATCACGCC – 3`<br>R: 5` - GAGTCCCGGATCACCGAACA - 3`	116	58	Present Study
EIF5A	F: 5` - CTTCACCTAGCTCCCTTGGC - 3`<br>R: 5` - TAAACCACAAGCAGCACCCA - 3`	123	58	Present Study

TPT1	F: 5` - GAGATCGCGGACGGGTTG - 3`<br>R: 5` - TTCAGCGGAGGCATTTCCAC - 3`	102	58	Present Study
NACA	F: 5` - ATTCCACCCAGGCAACCACACA - 3`<br>R: 5` - TGTAACCTGCCGAAGACCCAGT- 3`	143	58	Present Study
PPIA	F: 5` - ACTTCATCCTAAAGCATACGGGTC - 3`<br>R: 5` - CTCAGTCTTGGCAGTGCAGAT - 3`	101	58	Present Study
GABARAP	F: 5` - CTTGTGTTGCTCCCCTCGTC - 3`<br>R: 5` - TTGCAGACAGGGAAAAGCCC - 3`	153	58	Present Study
AURKA*	F: 5′- GGAGCCTTGGAGTTCTTTGC – 3′<br>R: 5'- CCTGGCTCCCTCTGTTACAA – 3′	134	58	[3]
BUB1*	F: 5` - GAGTGATATCTTCAGCTTGTG - 3`<br>R: 5` - AACAACCTGCTCAACATCAAC - 3`	122	58	[9]
SNAI1*	F: 5′- ACTATGCCGCGCTCTTTCCT – 3′<br>R: 5′ – GGTGGGGTTGAGGATCTCCG – 3′	162	58	Present Study

*Genes that were used as gene of interest for normalization by candidate reference genes

PRIMER EFFICIENCY CALCULATIONS

To calculate primer efficiency, the serial dilutions were performed twice using two different initial concentrations and two different log dilutions. The first serial dilution series was termed as "Broad Range" dilutions. The second serial dilution series was termed as "Narrow Range". The concentrations and log scale dilutions are shown below (NTC – no template control):

Broad Range			Narrow Range		
**Dilution Code**	**Concentration of cDNA (ng/ml)**	**Log scale dilution**	**Dilution Code**	**Concentration of cDNA (ng/ml)**	**Log Scale Dilution**
**B1**	20	1 : 1	**N1**	50	1 : 1
**B2**	2	1 : 10	**N2**	10	1 : 5
**B3**	0.2	1 : 100	**N3**	5	1 : 10
**B4**	0.02	1 : 1000	**N4**	1	1 : 50
**B5**	0.002	1 : 10,000	**N5**	0.1	1 : 500
**B6**	0.0002	1 : 100,000	**N6**	0.05	1 : 1000
**NTC**	NTC	NTC	**NTC**	NTC	NTC

As indicated in MIQE guidelines [10], the calibration curve (previously called as standard curve) was analyzed, which was prepared by plotting the log (cDNA conc) on x-axis against the Cq values on the y-axis. The samples were analyzed in triplicates for each dilution. The geomean (geometric mean) was used to estimate the "Central Cq" value from the triplicates. The geomean is a more accurate measure for averaging the Cq values than the normal mean/average because of the logarithmic nature of the Cq (as discussed in Additional file 3 (Extended Discussion) of our previous paper [3]).

The Slope, Amplification factor (E), Efficiency (F) and R^2 (coefficient of determination) for both series are shown in Additional Table 3 (next page). An efficiency of 100% (or amplification factor 2) symbolizes a perfect doubling of template cDNA (SK-BR-3) by the primer. R^2 represents how well the experimental data fit the regression line, that is how linear the data is. Linearity in turn gives a measure of the variability across assay replicates and whether the amplification efficiency is the same for different starting template copy numbers [11].

Note: The ideal recommended ranges for Efficiency (F) is from 90-110% while R^2 (coefficient of determination) is recommended to be ≥ 0.9800.

Additional Table 3. Primer efficiencies for the reference genes and genes of interest using calibration curves and two different serial dilution series

Gene Symbol	Broad Range				Narrow Range			
	Slope	Amplification factor (E)	Efficiency % (F)	R^2	Slope	Amplification factor (E)	Efficiency % (F)	R^2
ACTB	-3.250	2.031	103.09%	0.9997	-3.249	2.031	103.12%	0.9983
GAPDH	-3.535	1.918	91.81%	0.9852	-3.328	1.997	99.73%	0.9983
RPL13A	-2.974	2.168	116.89%	0.9984	-3.299	2.009	100.93%	0.9852
PGK1	-3.268	2.023	102.23%	0.9973	-3.104	2.099	109.98%	0.9850
HSP90AB1	-4.005	1.777	77.70%	0.9780	-3.137	2.083	108.34%	0.9990
RNA28S	-3.553	1.912	91.17%	0.9995	-3.428	1.958	95.76%	0.9847
RNA18S	-3.395	1.971	97.05%	0.9979	-3.187	2.056	105.96%	0.9812
PUM1	-3.505	1.929	92.89%	0.9946	-3.218	2.045	104.52%	0.9961
CCSER2	-2.178	2.876	187.57%	0.9921	-1.512	4.591	359.05%	0.9518
HNRNPL	-3.158	2.073	107.33%	0.9954	-3.315	2.003	100.28%	0.9819
PCBP1	-3.191	2.058	105.78%	0.9927	-3.316	2.004	100.35%	0.9921
SF3A1	-3.453	1.948	94.82%	0.9952	-3.276	2.019	101.95%	0.9815
DAD1	-3.297	2.011	101.05%	0.9980	-3.348	1.989	98.93%	0.9880
PSMB4	-3.378	1.977	97.73%	0.9983	-3.268	2.023	102.31%	0.9810
BSG	-3.409	1.965	96.49%	0.9929	-3.279	2.018	101.78%	0.9919
TUBA1B	-3.405	1.966	96.63%	0.9934	-3.133	2.085	108.54%	0.9802
RBX1	-3.392	1.971	97.14%	0.9980	-3.207	2.050	105.04%	0.9931

CFL1	-3.174	2.066	106.55%	0.9985	-3.299	2.009	100.94%	0.9955
UBC	-3.354	1.987	98.68%	0.9803	-3.128	2.088	108.79%	0.9971
PFN1	-3.266	2.024	102.41%	0.9985	-3.115	2.094	109.43%	0.9864
EIF5A	-3.540	1.916	91.64%	0.9977	-3.465	1.944	94.37%	0.9981
TPT1	-3.332	1.996	99.59%	0.9986	-3.088	2.108	110.79%	0.9913
NACA	-3.224	2.043	104.26%	0.9996	-3.065	2.119	111.99%	0.9868
PPIA	-3.423	1.959	95.96%	0.9983	-3.323	1.999	99.96%	0.9969
GABARAP	-2.472	2.539	153.86%	0.9964	-1.444	4.929	392.85%	0.9314
AURKA*	-3.298	2.010	101.03%	0.9980	-3.370	1.980	98.04%	0.9869
BUB1*	-3.176	2.065	106.47%	0.9994	-3.342	1.992	99.18%	0.9882
SNAI1*	-2.525	2.489	148.89%	0.9944	-3.112	2.096	109.57%	0.9804

*Genes that were used as gene of interest for normalization by candidate reference genes

Notes:

1) Some of the points amongst triplicates were removed only when they were identified and flagged by the ViiA 7 machine as definitive outliers/ no amplification/ experiment failed.

2) For *SF3A1, PUM1* and *AURKA* all the triplicates were “undetermined” for B6 dilution (broad range).

3) For *CCSER2* and *GABARAP,* the dilutions from B4-B6 (broad range) were removed while for *BUB1,* the dilutions from B5-B6 (broad range) and for *SNAI1,* dilutions from B6 (broad range) were removed from analysis due to flattening of curve and no change in Cq values.

4) For broad range series dilutions, *HSP90AB1* was outside the R^2 recommended limits while *CCSER2, GABARAP, RPL13A, HSP90AB1* and *SNAI1* were outside the Efficiency % recommended limits.

5) For narrow range series dilutions, *GABARAP* and *CCSER2* still remained outside the recommended Efficiency % limits and R^2 values while *TPT1* and *NACA* were marginally outside the recommended Efficiency % limits.

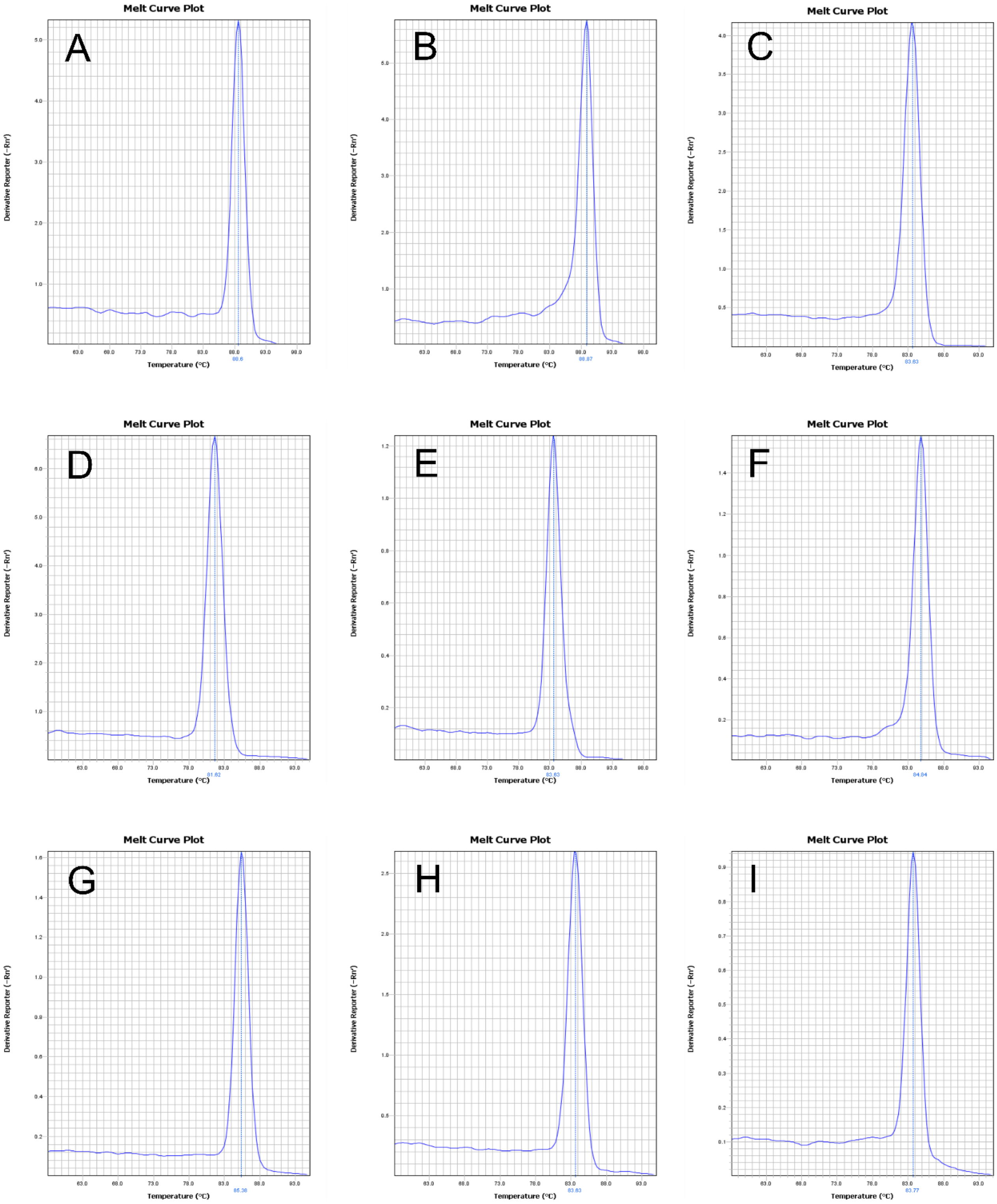

Additional Figure 1. The melting (dissociation) curve for the primers designed for the reference genes (**A**) *ACTB*; (**B**) *GAPDH*; (**C**) *RPL13A*; (**D**) *PGK1*; (**E**) *HSP90AB1*; (**F**) *RNA28S*; (**G**) *RNA18S*; (**H**) *PUM1* and (**I**) *CCSER2*. A single sharp amplicon peak indicates high amplification specificity of the primer pair.

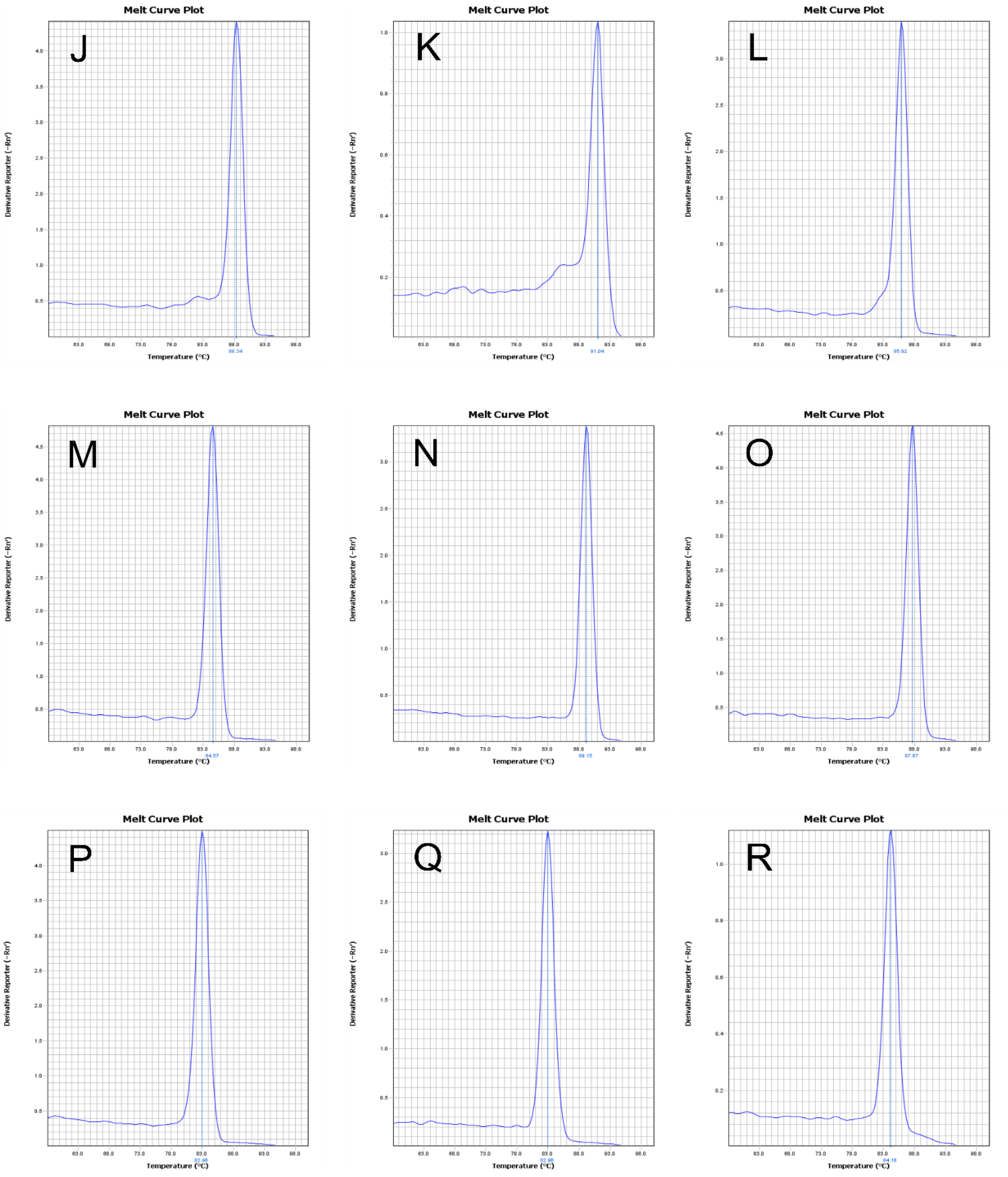

Additional Figure 2. The melting (dissociation) curve for the primers designed for the reference genes (**J**) *HNRNPL*; (**K**) *PCBP1*; (**L**) *SF3A1*; (**M**) *DAD1*; (**N**) *PSMB4*; (**O**) *BSG*; (**P**) *TUBA1B*; (**Q**) *RBX1* and (**R**) *CFL1*. A single sharp amplicon peak indicates high amplification specificity of the primer pair.

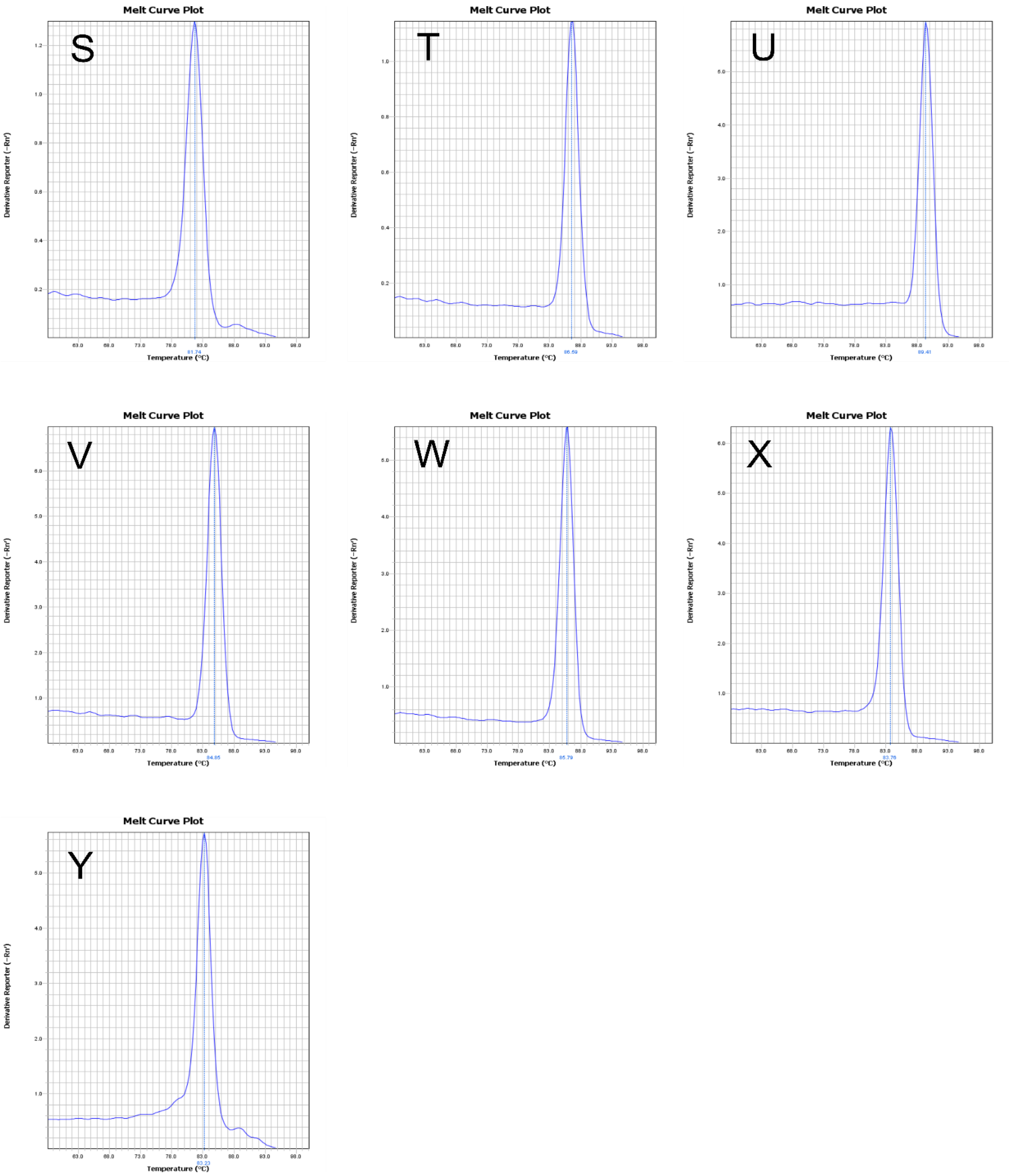

Additional Figure 3. The melting (dissociation) curve for the primers designed for the reference genes (**S**) *UBC*; (**T**) *PFN1*; (**U**) *EIF5A*; (**V**) *TPT1*; (**W**) *NACA*; (**X**) *PPIA* and (**Y**) *GABARAP*. A single sharp amplicon peak indicates high amplification specificity of the primer pair.

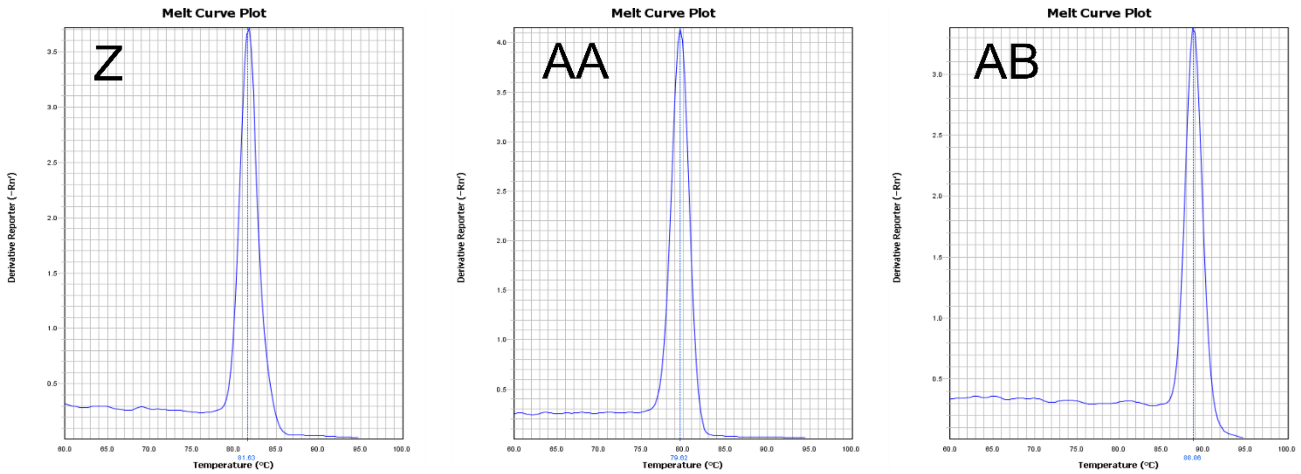

Additional Figure 4. The melting (dissociation) curve for the primers designed for the genes of interest (target genes) (**Z**) *AURKA*; (**AA**) *BUB1* and (**AB**) *SNAI1*. A single sharp amplicon peak indicates high amplification specificity of the primer pair.

REFERENCES

1. Liu LL, Zhao H, Ma TF, Ge F, Chen CS, Zhang YP. Identification of valid reference genes for the normalization of RT-qPCR expression studies in human breast cancer cell lines treated with and without transient transfection. PLoS One. 2015 Jan 24;10(1):e0117058. doi: 10.1371/journal.pone.0117058. PMID: 25617865; PMCID: PMC4305315.
2. Quiroz FG, Posada OM, Gallego-Perez D, Higuita-Castro N, Sarassa C, Hansford DJ, Agudelo-Florez P, López LE. Housekeeping gene stability influences the quantification of osteogenic markers during stem cell differentiation to the osteogenic lineage. Cytotechnology. 2010 Apr;62(2):109-20. doi: 10.1007/s10616-010-9265-1. Epub 2010 Apr 16. PMID: 20396946; PMCID: PMC2873986.
3. Jain N, Nitisa D, Pirsko V, Cakstina I. Selecting suitable reference genes for qPCR normalization: a comprehensive analysis in MCF-7 breast cancer cell line. BMC Mol Cell Biol. 2020 Sep 25;21(1):68. doi: 10.1186/s12860-020-00313-x. PMID: 32977762; PMCID: PMC7519550.
4. Balwierz A, Czech U, Polus A, Filipkowski RK, Mioduszewska B, Proszynski T, Kolodziejczyk P, Skrzeczynska-Moncznik J, Dudek W, Kaczmarek L, Kulig J, Pryjma J, Dembinska-Kiec A. Human adipose tissue stromal vascular fraction cells differentiate depending on distinct types of media. Cell Prolif. 2008 Jun;41(3):441-59. doi: 10.1111/j.1365-2184.2008.00531.x. Epub 2008 Apr 14. PMID: 18422701; PMCID: PMC6496779.
5. Kılıç Y, Çelebiler AÇ, Sakızlı M. Selecting housekeeping genes as references for the normalization of quantitative PCR data in breast cancer. Clin Transl Oncol. 2014 Feb;16(2):184-90. doi: 10.1007/s12094-013-1058-5. Epub 2013 May 30. PMID: 23720140.
6. Tilli TM, Castro Cda S, Tuszynski JA, Carels N. A strategy to identify housekeeping genes suitable for analysis in breast cancer diseases. BMC Genomics. 2016 Aug 15;17(1):639. doi: 10.1186/s12864-016-2946-1. PMID: 27526934; PMCID: PMC4986254.
7. Jo J, Choi S, Oh J, Lee SG, Choi SY, Kim KK, Park C. Conventionally used reference genes are not outstanding for normalization of gene expression in human cancer research. BMC Bioinformatics. 2019 May 29;20(Suppl 10):245. doi: 10.1186/s12859-019-2809-2. PMID: 31138119; PMCID: PMC6538551.
8. Maltseva DV, Khaustova NA, Fedotov NN, Matveeva EO, Lebedev AE, Shkurnikov MU, Galatenko VV, Schumacher U, Tonevitsky AG. High-throughput identification of reference genes for research and clinical RT-qPCR analysis of breast cancer samples. J Clin Bioinforma. 2013 Jul 22;3(1):13. doi: 10.1186/2043-9113-3-13. PMID: 23876162; PMCID: PMC3726509.
9. Yuan B, Xu Y, Woo JH, Wang Y, Bae YK, Yoon DS, Wersto RP, Tully E, Wilsbach K, Gabrielson E. Increased expression of mitotic checkpoint genes in breast cancer cells with chromosomal instability. Clin Cancer Res. 2006 Jan 15;12(2):405-10. doi: 10.1158/1078-0432.CCR-05-0903. PMID: 16428479.
10. Bustin SA, Benes V, Garson JA, Hellemans J, Huggett J, Kubista M, Mueller R, Nolan T, Pfaffl MW, Shipley GL, Vandesompele J, Wittwer CT. The MIQE guidelines: minimum information for publication of quantitative real-time PCR experiments. Clin Chem. 2009 Apr;55(4):611-22. doi: 10.1373/clinchem.2008.112797. Epub 2009 Feb 26. PMID: 19246619.
11. Bio-Rad Real Time PCR Applications Guide. 2006; pages 5-6. Bio-Rad Laboratories, Inc. http://www.bio-rad.com/webroot/web/pdf/lsr/literature/Bulletin_5279.pdf. Last accessed Oct 2020.
