# Supplementary material for: Identification of Novel Endogenous Controls for qPCR Normalization in SK-BR-3 Breast Cancer Cell Line
Source: Genes (Basel). 2021 Oct 17;12(10):1631. doi: 10.3390/genes12101631 (PMC8535678; doi:10.3390/genes12101631)
Supplement: Supplementary file 1 [file genes-12-01631-s001.zip › Supplementary Files/Jain et al. Supplementary File S2.pdf]

SUPPLEMENTARY FILE S2
GENE ONTOLOGY

Identification of novel endogenous controls for qPCR normalization in SK-BR-3 breast cancer cell line

Authors: Nityanand Jain*, Ingrida Mitre, Dina Nitisa, Valdis Pirsko and Inese Cakstina*

*** For Correspondence:**

Laboratory of Molecular Genetics
Institute of Oncology
Riga Stradins University
16 Dzirciema street
Riga
Latvia (LV-1007)

Email: nityapkl@gmail.com (NJ); inese.cakstina-dzerve@rsu.lv (IC)

INDEX

1) Additional Table 1 – Molecular Function (Gene Ontology) Analysis
2) Additional Table 2 – Cellular Component (Gene Ontology) Analysis
3) Additional Table 3 – Panther Functional Classification Analysis

Additional Table 1. Gene Ontology of Candidate Reference Genes ranked based on Fold Enrichment (Molecular Function)

GO ID	GO Term	No. of genes	Fold Enrichment	Raw P value	FDR
GO:0031625	Ubiquitin protein ligase binding	5	15.16 (+)	1.72E-05	2.74E-02
Genes: *RBX1, TUBA1B, HSPCB, UBC, GABARAP*					
GO:0003723	RNA binding	12	6.43 (+)	4.38E-08	2.31E-04
Genes: *SF3A1, TUBA1B, PUM1, HSPCB, RPL13A, TPT1, HNRNPL, PFN1, UBC, EIF5A, PPIA, PCBP1*					
GO:0008092	Cytoskeletal protein binding	7	6.36 (+)	7.30E-05	4.98E-02
Genes: *ACTB, GAPDH, HSPCB, PFN1, CFL1, CCSER2, GABARAP*					

No. of genes indicates the number of genes from the input selected candidate reference genes that are represented by the respective GO term. (+) indicates statistically significant over-representation of the GO term in the selected reference genes.

Notes:

1) Blue GO term indicates the most enriched molecular function while red GO term indicates the most significant molecular function.

Additional Table 2. Gene Ontology of Candidate Reference Genes ranked based on Fold Enrichment (Cellular Component)

GO ID	GO Term	No. of genes	Fold Enrichment	Raw P value	FDR
GO: 0097452	GAIT complex	2	> 100 (+)	1.74E-05	3.84E-03
***Genes:** GAPDH, RPL13A*					
GO:0035770	Ribonucleoprotein granule	4	14.56 (+)	1.56E-04	2.60E-02
***Genes:** ACTB, PUM1, HNRNPL, PCBP1*					
GO:0005925	Focal adhesion	6	13.04 (+)	5.04E-06	1.68E-03
***Genes:** ACTB, RPL13A, PFN1, CFL1, BSG, PPIA*					
GO:0070062	Extracellular exosome	14	6.05 (+)	3.82E-09	7.66E-06
***Genes:** ACTB, GAPDH, HSPCB, TPT1, HNRNPL, PFN1, PGK1, UBC, BSG, CFL1, NACA, PPIA, PSMB4, PCBP1*					
GO:0005829	Cytosol	16	2.73 (+)	1.14E-05	2.85E-03
***Genes:** GAPDH, RPL13A, RBX1, ACTB, PUM1, GABARAP, EIF5A, HSPCB, TPT1, PFN1, PGK1, UBC, CFL1, PPIA, PSMB4, PCBP1*					
GO:0005634	Nucleus	17	2.03 (+)	3.21E-04	4.92E-02
***Genes:** GAPDH, RPL13A, RBX1, SF3A1, ACTB, PUM1, EIF5A, HSPCB, TPT1, HNRNPL, PFN1, UBC, CFL1, NACA, PPIA, PSMB4, PCBP1*					

No. of genes indicates the number of genes from the input selected candidate reference genes that are represented by the respective GO term. (+) indicates statistically significant over-representation of the GO term in the selected reference genes.

Notes:

1) Blue GO term indicates the most enriched cellular component while red GO term indicates the most significant cellular component.

Additional Table 3. Panther Functional Classification Analysis (Supplement to Figure 2)

Class/Pathway	Number of Genes	Genes Included
Biological Process		
Cellular process	17	*BSG, CFL1, DAD1, EIF5A, GABARAP, HNRNPL, HSP90AB1, NACA, PFN1, PGK1, PSMB4, PUM1, RPL13A, RBX1, SF3A1, TUBA1B, UBC*
Metabolic process	11	*DAD1, EIF5A, GAPDH, GABARAP, HNRNPL, PCBP1, PGK1, PUM1, RBX1, SF3A1, UBC*
Biological regulation	8	*EIF5A, HSP90AB1, PCBP1, PFN1, PGK1, PUM1, RPL13A, SF3A1*
Response to stimulus	3	*BSG, HSP90AB1, GABARAP*
Developmental process	2	*BSG, PGK1*
Localization	2	*CFL1, NACA*
Locomotion	2	*BSG, CFL1*
Multicellular organismal process	2	*BSG, HSP90AB1*
Biological adhesion	1	*BSG*
Molecular Function		
Binding	16	*BSG, CFL1, EIF5A, GABARAP, HSP90AB1, NACA, PFN1, PGK1, PSMB4, PUM1, RPL13A, RBX1, SF3A1, TPT1, TUBA1B, UBC*
Catalytic activity	5	*DAD1, GAPDH, PGK1, PSMB4, RBX1*
Structural molecular activity	3	*ATCB, RPL13A, TUBA1B*
Translational regulator activity	1	*EIF5A*
Cellular Compartment		
Cell and cell part	17	*BSG1, CFL1, DAD1, GABARAP, GAPDH, HNRNPL, HSP90AB1, PFN1, PGK1, PPIA, PSMB4, PUM1, RBX1, RPL13A, SF3A1, TPT1, TUBA1B*
Organelle	10	*CFL1, DAD1, GABARAP, HNRNPL, PCPB1, PPIA, RBX1, RPL13A, SF3A1, UBC*
Organelle part	7	*CFL1, DAD1, GABARAP, PCBP1, PSMB4, RBX1, TUBA1B*
Protein-containing complex	6	*CFL1, DAD1, PSMB4, RPL13A, RBX1, SF3A1*
Membrane	5	*ACTB, BSG, DAD1, GABARAP, HSP90AB1*
Membrane-part	3	*ACTB, CFL1, TUBA1B*
Supramolecular complex	2	*TUBA1B, HSP90AB1*
Membrane-enclosed lumen	1	*PCBP1*

Protein Class		
Cytoskeletal protein	6	*ACTB, CFL1, GABARAP, PFN1, TPT1, TUBA1B*
Metabolite interconversion enzyme	3	*DAD1, GAPDH, PGK1*
Nucleic acid binding protein	3	*PCBP1, PUM1, SF3A1*
Translational protein	2	*EIF5A, RPL13A*
Chaperone	1	*HSP90AB1*
Gene-specific transcriptional regulator	1	*NACA*
Protein modifying enzyme	1	*RBX1*
Scaffold/adaptor protein	1	*BSG*
Pathway		
Cytoskeletal regulation by Rho GTPase	3	*ACTB, CFL1, PFN1*
Glycolysis	2	*GAPDH, PGK1*
Huntington disease	2	*ACTB, GAPDH*
Alzheimer disease-presenilin	1	*ACTB*
Cadherin signalling	1	*ACTB*
Gonadotropin releasing hormone receptor	1	*TUBA1B*
Inflammation mediated by chemokine and cytokine signalling	1	*ACTB*
Integrin signalling	1	*ACTB*
Nicotine acetylcholine receptor signalling	1	*ACTB*
Parkinson disease	1	*PSMB4*
Wnt Signalling	1	*ACTB*
