# Supplementary material for: Identification of Novel Endogenous Controls for qPCR Normalization in SK-BR-3 Breast Cancer Cell Line
Source: Genes (Basel). 2021 Oct 17;12(10):1631. doi: 10.3390/genes12101631 (PMC8535678; doi:10.3390/genes12101631)
Supplement: Supplementary file 1 [file genes-12-01631-s001.zip › Supplementary Files/Jain et al. Supplementary File S3.pdf]

SUPPLEMENTARY FILE S3
REPLICATE CULTURE ANALYSIS

Identification of novel endogenous controls for qPCR normalization in SK-BR-3 breast cancer cell line

Authors: Nityanand Jain*, Ingrida Mitre, Dina Nitisa, Valdis Pirsko and Inese Cakstina*

*** For Correspondence:**

Laboratory of Molecular Genetics
Institute of Oncology
Riga Stradins University
16 Dzirciema street
Riga
Latvia (LV-1007)

Email: nityapkl@gmail.com (NJ); inese.cakstina@rsu.lv (IC)

INDEX

1) Section 1: Descriptive Analysis for Culture S1 and S2
2) Section 2: Coefficient of Variation (*CV*%)
3) Section 3: geNorm Analysis for Culture S1 and S2
4) Section 4: BestKeeper Analysis for Culture S1 and S2
5) Section 5: Need for Re-analysis due to Removal for genes
6) Section 6: NormFinder Analysis for Culture S1 and S2
7) Section 7: Comparative ΔCt Analysis in Culture S1 and S2
8) Section 8: RefFinder Analysis for Culture S1 and S2
9) Section 9: Final Cumulative Ranking of Reference Genes for SK-BR-3 cell line
10) Section 10: References

Section 1 : Descriptive Analysis for Culture S1 and S2

All candidate reference genes showed almost identical expression levels in both cultures S1 and S2. *RNA28S* and *RNA18S* were found to be highly expressed in both cultures (Additional Figure 1). Both the genes were amplified almost nine cycles earlier than any other gene in both cultures. *TPT1, GAPDH, ACTB* and *PPIA* were the next to be amplified in both cultures (Additional Figure 1a and 1b). The lowest expression was found for *GABARAP* (Additional Figure 1d). This is interesting since according to TCGA data, *GABARAP* was rather expected to showcase moderate expression.

The largest variation between Cq values was shown by *TUBA1B* in both cultures (S.D = 1.05 and 0.90 respectively). It was followed by *RNA28S* (S.D = 0.99) in culture S1 while *RNA18S* followed in culture S2 (S.D = 0.78). The least variation in culture S1 was shown by *EIF5A* (S.D = 0.34) closely followed by *UBC* and *CFL1* (S.D = 0.35 each) while in culture S2, *UBC* (S.D = 0.25) showed the least variation which was followed by *RPL13A* and *NACA* (S.D = 0.35 each).

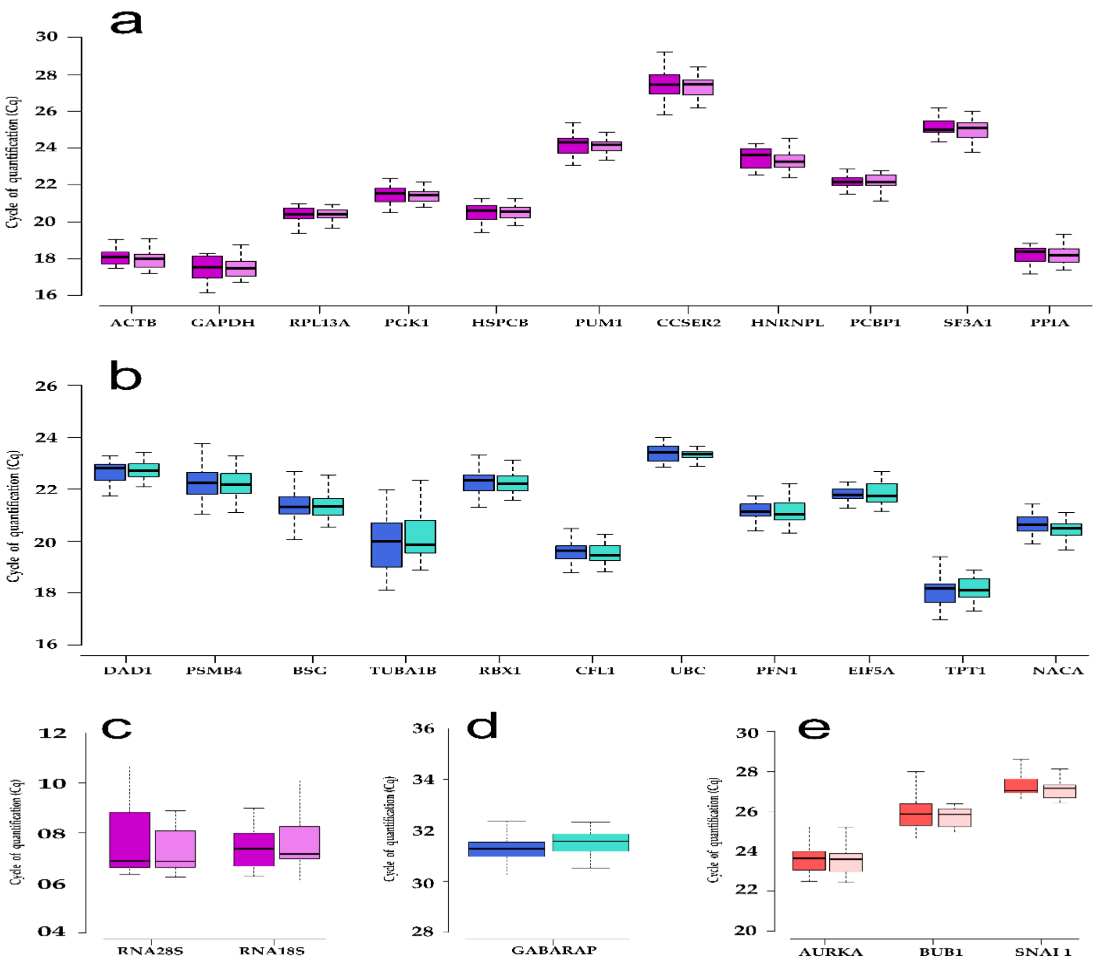

Additional Figure 1. Expression of the selected candidate reference genes (**a-d**) and genes of interest (**e**) as obtained from RT-qCPR in terms of Cq values. (**a**) Expression of traditional candidate reference genes in culture S1 (magenta) and S2 (light pink); (**b**) Expression of novel candidate reference genes in culture S1 (dark blue) and S2 (light blue); (**c**) Expression of traditional candidate reference genes (*RNA28S* and *RNA18S*) in culture S1 (magenta) and S2 (light pink); (**d**) Expression of novel candidate reference gene (*GABARAP*) in culture S1 (dark blue) and S2 (light blue); (**e**) Expression of genes of interest in culture S1 (dark red) and S2 (light red). *HSPCB* represents *HSP90AB1*.

Although the MIQE guidelines [1] introduced and defined the term "reference gene/s", they remain rather silent on the acceptable (or ideal) range of Cq values for reference gene. Other authors meanwhile suggest to select a candidate reference gene with Cq in the range of 15-30 cycles [2-4]. < 15 cycles have been associated with very high expression while > 30 cycles has been associated with very low expression. Keeping in mind these limits, we removed *GABARAP*, *RNA18S* and *RNA28S* from further analysis. *CCSER2* was also removed due to expression very close to the cut-off limits.

Section 2: Coefficient of Variation (CV%)

CV% is calculated as the ratio of standard deviation (S.D) and mean of linearized Cq value (2^{-Cq}) and expressed in percentage (by multiplying with 100). In general, lower the *CV*%, the more stable the reference gene and visa-versa. Hellemans et al. [5-6] suggest that the mean *CV*% values should remain below 25% for a stably expressed reference gene in a homogenous panel while it should be below 50% for heterogenous panel.

Additional Figure 2 shows the mean *CV*% for all biological replicates for the reference genes in both cultures. A summary of the mean *CV*% is presented in Additional Table 1. *TUBA1B* (*CV*% = 57.841) violated the cutoff (*CV*% < 50%) in culture S1 whilst also showing the highest variation in culture S2 (*CV*% = 41.796) and was hence, removed from further analysis.

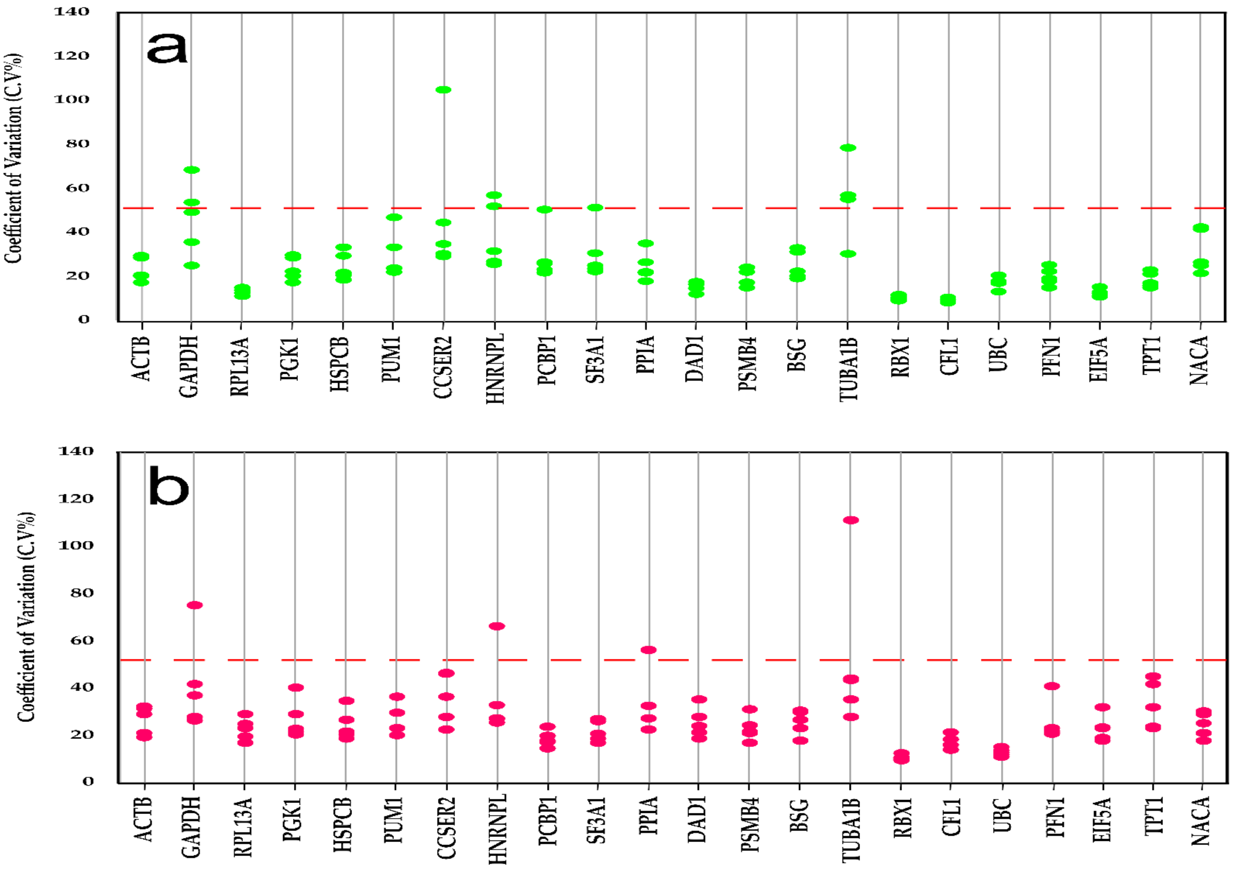

Additional Figure 2. Coefficient of variation (*CV*%) for all candidate reference genes in both cultures for all biological replicates (shown as individual colored dot). (**a**) *CV*% of candidate reference genes in culture S1 (blue

dots); (**b**) *CV%* of candidate reference genes in culture S2 (magenta dots). The red dashed line depicts the acceptable range of *CV%* (≤ 50%) for heterogenous tissue/samples. *HSPCB* represents *HSP90AB1*.

Additional Table 1. Mean Linearized Coefficient of Variance (*CV%*) for SK-BR-3 cultures S1 and S2

Gene	Mean Linearized *CV%* (SK-BR-3)	Mean Linearized *CV%* (S1)	Mean Linearized *CV%* (S2)
ACTB	24.278	22.512	25.119
GAPDH	35.956	41.344	36.007
RPL13A	16.356	12.754	21.669
PGK1	22.311	22.879	25.012
HSP90AB1	21.656	23.683	22.967
RNA28S	34.089	31.493	27.824
RNA18S	41.973	43.176	45.384
PUM1	23.967	27.890	24.368
CCSER2	32.883	39.299	32.912
HNRNPL	30.682	34.658	31.260
PCBP1	21.776	27.285	17.887
SF3A1	24.495	28.189	20.868
PPIA	25.070	23.659	29.890
DAD1	18.590	15.056	23.940
PSMB4	19.377	18.782	21.909
BSG	23.168	24.108	24.316
TUBA1B	54.754	57.841	41.796
RBX1	9.237	10.553	10.221
CFL1	13.782	9.394	16.907
UBC	14.855	17.067	12.460
PFN1	20.548	19.532	23.765
EIF5A	17.428	12.609	21.863
TPT1	23.117	18.079	30.454
NACA	24.478	29.336	23.415
GABARAP	27.760	22.345	39.208
AURKA*	45.785	45.642	45.873
BUB1*	51.355	53.081	49.629
SNAI1*	20.047	26.449	15.605

* Genes that were used as genes of interest (GOI) in the present study

Notes: Blue marked genes were eliminated due to very high/very low expression levels in terms of Cq value. Red marked gene was removed due to very high *CV*% (>50% for heterogenous tissue).

Section 3: geNorm Analysis for Culture S1 and S2

In our analysis until now, 5 out of 25 candidate reference genes (*RNA18S, RNA28S, GABARAP, CCSER2* and *TUBA1B*) have been removed from analysis due to various reasons detailed above. geNorm calculates *M* value (stability value) for each candidate gene based on pairwise comparisons by employing stepwise exclusion method [7]. Recommended cutoff value is set at < 1 for heterogenous tissue (0.5 for homogenous tissue) [5-6]. All 20-reference genes were found to be below the recommended cutoff value in both cultures. In culture S1 (Additional Figure 3a), *CLF1, EIF5A, RBX1* and *DAD1* were ranked as the most stable genes while for culture S2 (Additional Figure 3b), *DAD1, HSP90AB1, PUM1* and *PGK1* were the most stable genes.

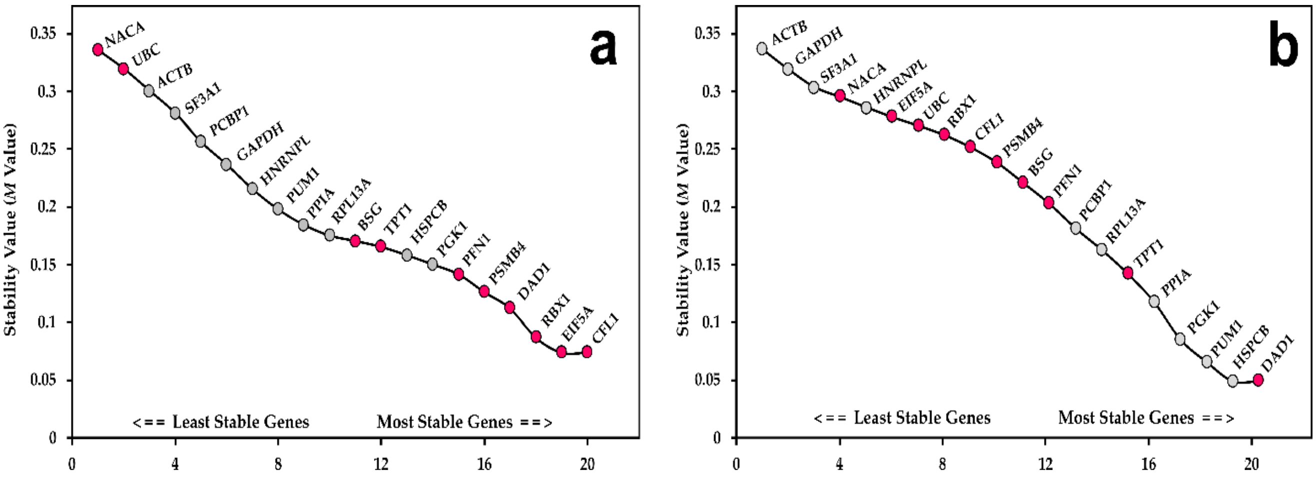

Additional Figure 3. geNorm analysis of candidate reference genes in (**a**) culture S1 and (**b**) culture S2 ranked according to *M* value. Pink circles indicate novel candidate reference genes while Grey circles indicate traditional candidate reference genes. *HSPCB* represents *HSP90AB1*.

Determination of the Optimal Number of Reference Genes Required for Normalization

geNorm can be further used to estimate optimal number of genes required for accurate normalization of expression data [7]. Recommended cutoff is $V_n/V_{n+1} < 0.15$ where V_n represents the number of genes suitable for normalization and addition of more genes would not affect normalization results. However, as good practice, it has been suggested in cases of $V_n = 2$, a third reference gene should also be considered and added in the panel [5-6]. For both cultures S1 and S2, we found that $V_{2/3}$ was less than 0.15 (Additional Figure 4), hence we considered and recommend use of three reference genes for normalization in SK-BR-3 cell line.

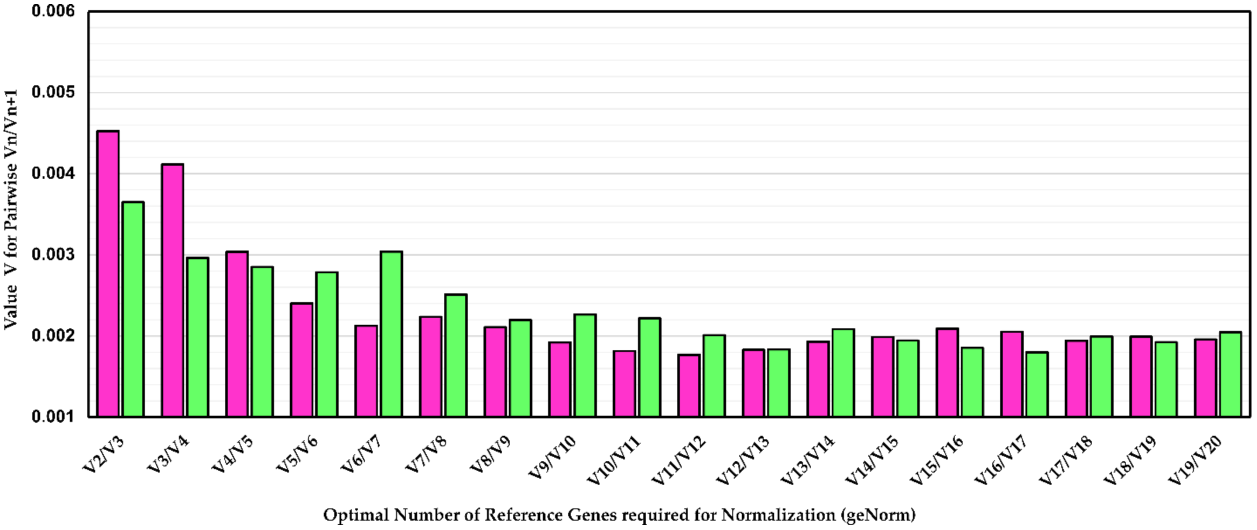

Additional Figure 4. Determination of optimal number of reference genes needed for normalization (geNorm) for both cultures (culture S1 is shown in pink bars while culture S2 is shown in green bars) using pairwise Vn/n + 1 analysis. The recommended cutoff is the lowest Vn/n + 1 below the threshold of 0.15. In the present study, both the cultures had V2/3 less than 0.15, indicating addition of a third reference gene would not affect normalization results.

Section 4: BestKeeper Analysis for Culture S1 and S2

BestKeeper analyzes the expression stability of reference genes using crossing points (C.P) to decide whether the genes are differentially expressed under the applied conditions or not [8]. All genes were found to be within recommended limits (S.D ± C.P < 1) in both cultures (Additional Figure 5). In culture S1, *CFL1* was found to be the most stable gene while *RBX1* was the most stable gene in culture S2. In both cultures, *GAPDH* was found to have least stable expression.

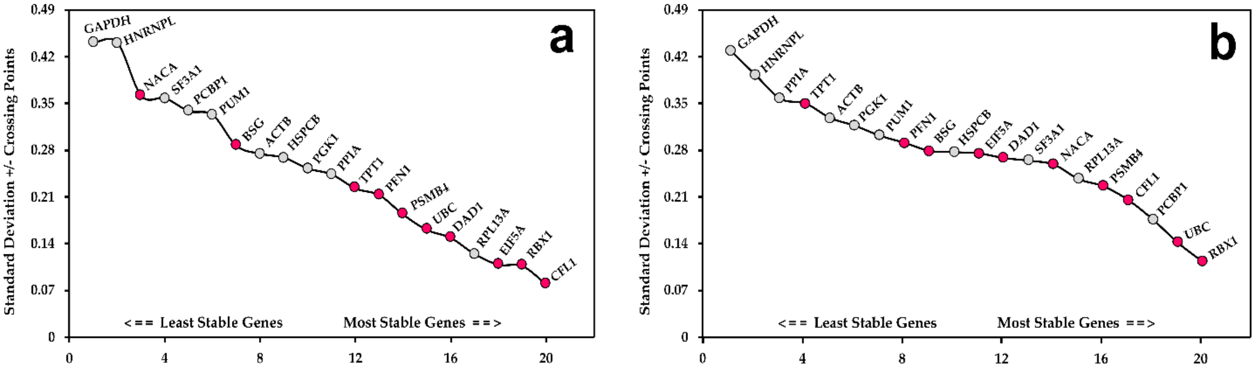

Additional Figure 5. BestKeeper results for standard deviation with crossing points (S.D ± C.P) on the Y axis with the selected candidate genes on the X-axis for (**a**) culture S1 and (**b**) culture S2. The most stable genes are considered to have a S.D as close as possible to 0 but not greater than 1. Pink circles indicate novel candidate reference genes while Grey circles indicate traditional candidate reference genes. *HSPCB* represents *HSP90AB1*.

The analysis was further used to estimate the expression correlation of the candidate reference genes (Additional Figure 6) for both cultures. The algorithm uses Pearson's correlation (r) to estimate the correlation between genes. In culture S1, a high positive correlation (top 5) was found between (r > 0.5)

HSP90AB1-PGK1, PUM1-HNRNPL, DAD1-PPIA, PUM1-PGK1 and *HNRNPL-HSP90AB1* (r = 0.942, 0.893, 0.892. 0.882 and 0.881 respectively). Similarly, in culture S2, *PUM1-HSP90AB1, TPT1-PUM1, DAD1-PPIA, TPT1-DAD1* and *DAD1-HSP90AB1* (r = 0.912, 0.899, 0.897, 0.896 and 0.895 respectively) were found to show a high positive correlation (top 5).

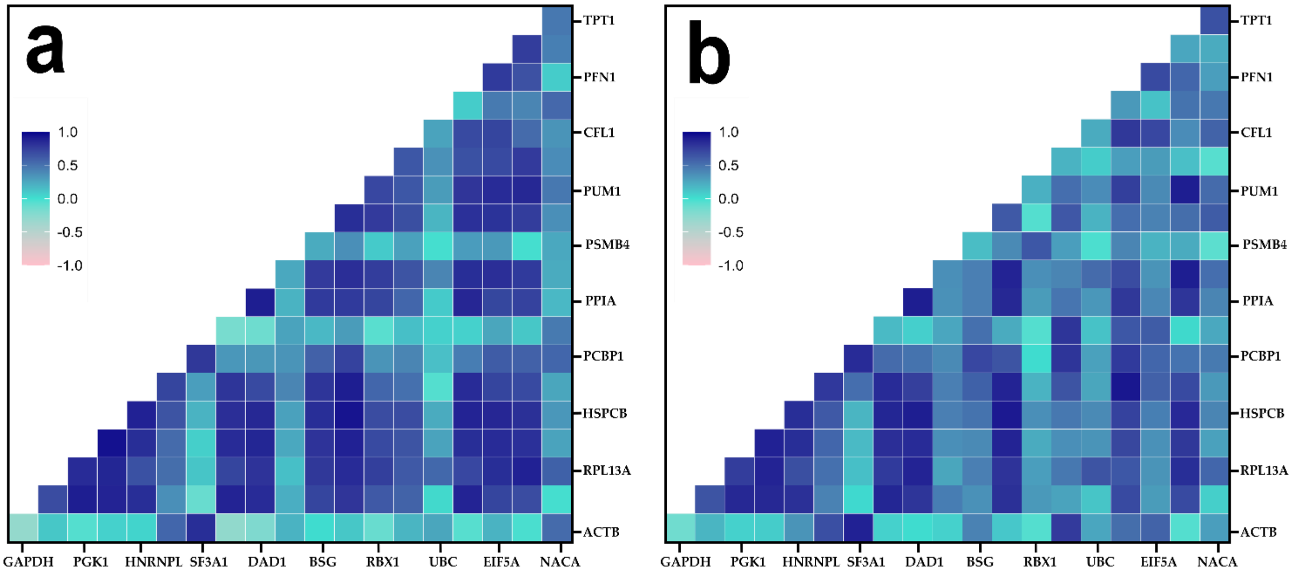

Additional Figure 6. Pearson correlation of the pairwise gene expression stability as obtained from BestKeeper algorithm. The correlation was assessed for all candidate genes in both (**a**) culture S1 and (**b**) culture S2. The darker the blue, the higher the positive correlation between the genes as seen in the legend. *HSPCB* represents *HSP90AB1*.

Further, the BestKeeper algorithm can be used to calculate ranking of reference gene (stability) in terms of fold change and correlation to the BestKeeper index as shown in Additional Table 2 and 3.

Additional Table 2. Ranking of reference genes based on BestKeeper analysis for fold change

Gene	Rank		S.D ± x-fold		Minimum x-fold		Maximum x-fold	
	S1	S2	S1	S2	S1	S2	S1	S2
ACTB	13	16	1.210	1.257	-1.322	-1.382	1.304	1.388
GAPDH	19	20	1.358	1.348	-1.690	-1.465	1.512	1.948
RPL13A	4	6	1.091	1.178	-1.153	-1.339	1.175	1.298
PGK1	11	15	1.192	1.246	-1.319	-1.281	1.263	1.571
HSP90AB1	12	11	1.205	1.211	-1.296	-1.293	1.354	1.452
PUM1	15	14	1.260	1.233	-1.290	-1.256	1.610	1.446
HNRNPL	19	19	1.358	1.314	-1.400	-1.323	1.539	1.988
PCBP1	16	3	1.265	1.128	-1.290	-1.285	1.803	1.279
SF3A1	17	8	1.282	1.201	-1.306	-1.307	1.780	1.196

PPIA	10	18	1.184	1.281	-1.338	-1.397	1.420	1.787
DAD1	5	9	1.109	1.204	-1.251	-1.343	1.136	1.425
PSMB4	7	5	1.136	1.169	-1.361	-1.334	1.260	1.348
BSG	14	12	1.220	1.213	-1.212	-1.479	1.296	1.230
RBX1	2	1	1.077	1.079	-1.120	-1.108	1.101	1.170
CFL1	1	4	1.057	1.152	-1.132	-1.279	1.113	1.231
UBC	6	2	1.118	1.101	-1.318	-1.181	1.191	1.195
PFN1	8	13	1.159	1.223	-1.299	-1.218	1.261	1.658
EIF5A	3	10	1.078	1.210	-1.143	-1.307	1.204	1.398
TPT1	9	17	1.168	1.275	-1.186	-1.375	1.325	1.411
NACA	18	7	1.285	1.196	-1.331	-1.370	1.376	1.253

The BestKeeper algorithm can be used to tabulate the BestKeeper Index (BI). The BI specific for the respective samples is calculated as the geometric mean of its candidate HKGs C.P (crossing point) values [8]. Once the BI is formulated, then correlation between each candidate HKG and the index is calculated, describing the relation between the index and the contributing candidate HKG (Additional Table 3). It is desirable to have HKG with very high/ strong levels of correlation with the index.

Additional Table 3. Correlation index (BestKeeper index vs HKG) for both cultures

**Gene**	**Culture S1**		**Culture S2**	
	**r**	***P* value**	**r**	***P* value**
ACTB	0.209	0.736	0.199	0.748
GAPDH	0.711	0.178	0.808	0.098
RPL13A	0.870	0.055	0.840	0.075
PGK1	0.912	0.031	0.915	0.029
HSP90AB1	0.971	0.006	0.927	0.023
PUM1	0.998	0.000	0.944	0.016
HNRNPL	0.955	0.012	0.973	0.005
PCBP1	0.881	0.048	0.942	0.017
SF3A1	0.664	0.222	0.448	0.450
PPIA	0.655	0.230	0.935	0.020
DAD1	0.693	0.195	0.920	0.027
PSMB4	0.885	0.046	0.668	0.218
BSG	0.916	0.029	0.759	0.137

RBX1	0.846	0.071	0.561	0.325
CFL1	0.726	0.165	0.734	0.158
UBC	-0.490	0.402	0.502	0.389
PFN1	0.760	0.136	0.918	0.028
EIF5A	0.928	0.023	0.738	0.154
TPT1	0.940	0.017	0.868	0.057
NACA	0.268	0.663	0.505	0.386

Interpretation for r ranges: 0 is no association; 0 – 0.25 is negligible association; 0.50 – 0.75 is moderate association; 0.75 – 1.00 is very strong association and 1.00 is perfect association. Blue highlight shows weak/moderate correlation while red highlight shows negative correlation.

Based on Additional Table 3, genes that were not found to have very strong correlation with the index (genes marked in blue; $r < 0.75$) were identified. Genes with negative correlation are marked in red. We identified 5 candidate reference genes that were weakly correlated in both the cultures S1 and S2 – *ACTB, SF3A1, CFL1, UBC* and *NACA*. These genes were hence, removed from further analysis.

Section 5: Need for Re-analysis due to Removal of genes

Due to the removal of these 5 candidate reference genes, geNorm and BestKeeper were used again to re-evaluate the changes induced in the ranking by both algorithms. This was needed because of the relative approach used by both these algorithms which gets affected by the presence or absence of individual reference genes. Additional Tables 4 and 5 briefly summarizes the re-analyzed rankings. geNorm Vn/n+1 analysis revealed that still 2 reference genes would be sufficient for successful normalization of genes of interest (V2/3 = 0.00452 and 0.00364 respectively for culture S1 and S2).

Additional Table 4. Re-evaluation of geNorm Analysis after removal of reference genes candidates

**Culture S1**			**Culture S2**		
**Gene**	***M* Value**	**Ranking**	**Gene**	***M* Value**	**Ranking**
RBX1	0.095	1	***HSP90AB1***	0.056	1
EIF5A	0.095	1	***DAD1***	0.056	1
DAD1	0.122	2	***PUM1***	0.073	2
PSMB4	0.131	3	***PGK1***	0.092	3
PFN1	0.142	4	***PPIA***	0.123	4
PGK1	0.147	5	***TPT1***	0.147	5
HSP90AB1	0.153	6	***RPL13A***	0.168	6
TPT1	0.161	7	***PCBP1***	0.186	7
BSG	0.165	8	***PFN1***	0.207	8
RPL13A	0.172	9	***HNRNPL***	0.222	9
PPIA	0.181	10	***BSG***	0.239	10
PUM1	0.195	11	***PSMB4***	0.253	11
HNRNPL	0.212	12	***RBX1***	0.267	12
GAPDH	0.234	13	***EIF5A***	0.278	13
PCBP1	0.255	14	***GAPDH***	0.294	14

Additional Table 5. Re-evaluation of BestKeeper Analysis after removal of reference genes candidates

Culture S1			Culture S2		
**Gene**	**S.D ± C.P**	**Ranking**	**Gene**	**S.D ± C.P**	**Ranking**
RBX1	0.107	1	***RBX1***	0.110	1
EIF5A	0.109	2	***PCBP1***	0.174	2
RPL13A	0.125	3	***PSMB4***	0.225	3
DAD1	0.149	4	***RPL13A***	0.236	4
PSMB4	0.184	5	***DAD1***	0.268	5
PFN1	0.213	6	***EIF5A***	0.275	6
TPT1	0.223	7	***HSP90AB1***	0.277	7
PPIA	0.244	8	***BSG***	0.279	8
PGK1	0.253	9	***PFN1***	0.291	9
HSP90AB1	0.269	10	***PUM1***	0.302	10
BSG	0.287	11	***PGK1***	0.317	11
PUM1	0.333	12	***TPT1***	0.351	12
PCBP1	0.339	13	***PPIA***	0.358	13
HNRNPL	0.441	14	***HNRNPL***	0.394	14
GAPDH	0.442	15	***GAPDH***	0.431	15

Section 6: NormFinder Analysis for Culture S1 and S2

NormFinder estimates the overall variation in gene expression for each gene and delivers a stability value that identifies not only the most stable gene but also the best control gene [9]. In principle, lower the group standard deviation, higher the expression stability of the gene. As shown in Additional Table 6, in both culture S1 and S2, we found that *HSP90AB1* was the most stably expressed gene (S.D = 0.04 and 0.07 respectively). Further, we used the algorithm to analyze the top 5 gene pairs for normalization (Additional Table 7). *PGK1-BSG* and *HSP90AB1-PSMB4* were found to be the most stable gene pairs in culture S1 while *PGK1-DAD1* was found to be most stable in culture S2.

Additional Table 6. Ranking of Genes based on NormFinder Analysis for both cultures S1 and S2

Culture S1			Culture S2		
**Gene**	**Group S.D**	**Ranking**	**Gene**	**Group S.D**	**Ranking**
HSP90AB1	0.04	1	***HSP90AB1***	0.07	1
PGK1	0.05	2	***PUM1***	0.08	2
PSMB4	0.10	3	***PGK1***	0.10	3
BSG	0.10	3	***DAD1***	0.11	4
PFN1	0.12	4	***PCBP1***	0.16	5
TPT1	0.13	5	***PFN1***	0.19	6
DAD1	0.16	6	***PPIA***	0.19	6
EIF5A	0.16	6	***RPL13A***	0.21	7
RBX1	0.18	7	***TPT1***	0.23	8
PUM1	0.19	8	***PSMB4***	0.24	9

RPL13A	0.19	8	***HNRNPL***	0.25	10
PPIA	0.20	9	***BSG***	0.29	11
HNRNPL	0.26	10	***RBX1***	0.29	11
GAPDH	0.35	11	***EIF5A***	0.31	12
PCBP1	0.36	12	***GAPDH***	0.36	13

Additional Table 7. Top 5 Pair of Genes ranked based on Group Standard deviation (S.D) by NormFinder

**Culture S1**			**Culture S2**		
**Gene Pair**	**Group S.D**	**Ranking**	**Gene Pair**	**Group S.D**	**Ranking**
PGK1-BSG	0.03	1	***PGK1-DAD1***	0.01	1
HSP90AB1-PSMB4	0.03	1	***HSP90AB1-PUM1***	0.03	2
PGK1-PFN1	0.05	2	***PGK1-PCBP1***	0.04	3
PGK1-PSMB4	0.05	2	***HSP90AB1-DAD1***	0.04	3
PSMB4-BSG	0.05	2	***HSP90AB1-PCBP1***	0.05	4
PFN1-PSMB4	0.04	3	***DAD1-PCBP1***	0.51	5
PFN1-BSG	0.59	4	***PGK1-HPSCB***	0.78	6
PGK1-HSP90AB1	0.60	5	***PGK1-PUM1***	0.89	7

Section 7: Comparative ΔCt Analysis in Culture S1 and S2

Another frequently used algorithm, Comparative ΔCt compares the relative expression of pairs of candidate reference genes within each sample to identify and rank the most stable genes [10]. A stable gene should be strongly expressed, should display minimum fluctuations and is independent of expression of other genes. Hence, lower the standard deviation, higher the expression stability of the gene. The results from comparative ΔCt showed *PGK1-HSP90AB1* being the most stable gene in culture S1 and sharing the position with *HSP90AB1-PUM1* in culture S2 (Additional Table 8).

Additional Table 8. Ranking of Genes based on Comparative ΔCt Analysis for both cultures S1 and S2

**Culture S1**			**Culture S2**		
**Gene**	**Average S.D**	**Ranking**	**Gene**	**Average S.D**	**Ranking**
PGK1	0.19	1	***HSP90AB1***	0.22	1
HSP90AB1	0.20	2	***PUM1***	0.22	1
PSMB4	0.21	3	***DAD1***	0.23	2
PFN1	0.21	3	***PGK1***	0.23	2
BSG	0.21	3	***PCBP1***	0.26	3

TPT1	0.22	4	***PPIA***	0.28	4
DAD1	0.23	5	***PFN1***	0.29	5
EIF5A	0.23	5	***RPL13A***	0.29	5
RBX1	0.24	6	***TPT1***	0.30	6
RPL13A	0.26	7	***PSMB4***	0.32	7
PPIA	0.26	7	***HNRNPL***	0.32	7
PUM1	0.27	8	***BSG***	0.34	8
HNRNPL	0.31	9	***RBX1***	0.35	9
GAPDH	0.38	10	***EIF5A***	0.36	10
PCBP1	0.39	11	***GAPDH***	0.40	11

Section 8: RefFinder Analysis for Culture S1 and S2

Finally, RefFinder [11] is an online free web-based tool (https://www.heartcure.com.au/reffinder/) which measures the geometric mean of attributed weights by NormFinder, geNorm, BestKeeper and Comparative ΔCt to generate an overall final ranking (Additional Table 9). As with other algorithms, lower the geometric mean by RefFinder, the higher the expression stability of the gene. Our analysis revealed *RBX1, PGK1* and *EIF5A* as the top three genes in culture S1 while *HSP90AB1, DAD1* and *PUM1* in culture S2.

Additional Table 9. Ranking of genes based on RefFinder Analysis for both cultures S1 and S2

**Culture S1**			**Culture S2**		
**Gene**	**Geomean**	**Ranking**	**Gene**	**Geomean**	**Ranking**
RBX1	3.00	1	***HSP90AB1***	1.63	1
PGK1	3.22	2	***DAD1***	2.78	2
EIF5A	3.36	3	***PUM1***	3.31	3
HSP90AB1	3.44	4	***PCBP1***	4.47	4
PSMB4	3.66	5	***PGK1***	4.79	5
DAD1	4.92	6	***RPL13A***	6.51	6
PFN1	4.95	7	***RBX1***	6.85	7
BSG	6.67	8	***PPIA***	7.23	8
TPT1	6.70	9	***PFN1***	7.64	9
RPL13A	7.58	10	***PSMB4***	7.75	10
PPIA	10.38	11	***TPT1***	8.74	11
PUM1	11.47	12	***BSG***	10.61	12
HNRNPL	13.24	13	***EIF5A***	11.33	13
GAPDH	14.24	14	***HNRNPL***	11.41	14
PCBP1	14.47	15	***GAPDH***	15.00	15

Section 9: Final Cumulative Ranking of Reference Genes for **SK-BR-3** *(combined S1 and S2) cell line after removal of genes*

Finally, after the removal of 10 reference gene candidates, a cumulative ranking combining the two replicate cultures was performed as summarized below.

Additional Table 10. Ranking of Candidate Reference Genes for SK-BR-3 cell line (cultures S1 and S2 combined)

NormFinder			geNorm			Comparative ΔCt		
**Candidate Gene**	**Group S.D**	**Rank**	**Candidate Gene**	**Stability value M**	**Rank**	**Candidate Gene**	**Average S.D**	**Rank**
HSP90AB1	0.13	1	*PGK1*	0.19	1	*HSP90AB1*	0.31	1
DAD1	0.18	2	*HSP90AB1*	0.19	1	*DAD1*	0.33	2
PUM1	0.20	3	*DAD1*	0.22	2	*PGK1*	0.34	3
PGK1	0.20	3	*RPL13A*	0.23	3	*PUM1*	0.34	3
PFN1	0.20	3	*PUM1*	0.24	4	*RPL13A*	0.35	4
RPL13A	0.21	4	*TPT1*	0.25	5	*PFN1*	0.35	4
PPIA	0.24	5	*PPIA*	0.27	6	*PPIA*	0.36	5
TPT1	0.27	6	*PFN1*	0.28	7	*TPT1*	0.38	6
HNRNPL	0.29	7	*HNRNPL*	0.30	8	*HNRNPL*	0.39	7
CFL1	0.31	8	*GAPDH*	0.31	9	*CFL1*	0.41	8
EIF5A	0.34	9	*CFL1*	0.33	10	*EIF5A*	0.44	9
PCBP1	0.36	10	*EIF5A*	0.34	11	*GAPDH*	0.45	10
GAPDH	0.36	10	*PCBP1*	0.36	12	*PCBP1*	0.45	10
BSG	0.39	11	*BSG*	0.38	13	*BSG*	0.46	11
RBX1	0.42	12	*RBX1*	0.39	14	*RBX1*	0.47	12
**BestKeeper**			**Correlation with BestKeeper Index**			**RefFinder**		
**Candidate Gene**	**S.D ± C.P**	**Rank**	**Candidate Gene**	**Correlation r**	**Rank**	**Candidate Gene**	**Geomean**	**Rank**
RPL13A	0.302	1	*HSP90AB1*	0.953	1	*HSP90AB1*	1.68	1
CFL1	0.305	2	*PUM1*	0.931	2	*DAD1*	2.63	2
EIF5A	0.327	3	*PGK1*	0.913	3	*PGK1*	3.22	3
DAD1	0.332	4	*HNRNPL*	0.905	4	*RPL13A*	3.31	4
PFN1	0.339	5	*DAD1*	0.892	5	*PUM1*	5.07	5
PCBP1	0.341	6	*PPIA*	0.890	6	*PFN1*	5.89	6
RBX1	0.347	7	*PFN1*	0.880	7	*CFL1*	6.85	7
HSP90AB1	0.374	8	*GAPDH*	0.863	8	*TPT1*	7.87	8
PGK1	0.378	9	*RPL13A*	0.856	9	*PPIA*	8.01	9
TPT1	0.388	10	*TPT1*	0.850	10	*EIF5A*	8.12	10
PUM1	0.400	11	*BSG*	0.773	11	*HNRNPL*	10.05	11
PPIA	0.410	12	*EIF5A*	0.692	12	*PCBP1*	10.50	12
BSG	0.438	13	*CFL1*	0.688	13	*GAPDH*	12.37	13
HNRNPL	0.489	14	*PCBP1*	0.669	14	*RBX1*	12.40	14
GAPDH	0.521	15	*RBX1*	0.589	15	*BSG*	13.74	15

Section 10: References

1. Bustin SA, Benes V, Garson JA, Hellemans J, Huggett J, Kubista M, Mueller R, Nolan T, Pfaffl MW, Shipley GL, Vandesompele J, Wittwer CT. The MIQE guidelines: minimum information for publication of quantitative real-time PCR experiments. Clin Chem. 2009 Apr;55(4):611-22. doi: 10.1373/clinchem.2008.112797. Epub 2009 Feb 26. PMID: 19246619.

2. Picard C, Silvy M, Gabert J. Overview of Real-Time RT-PCR Strategies for Quantification of Gene Rearrangements in the Myeloid Malignancies. In: Iland H, Hertzberg M, Marlton P, editors. Myeloid leukemia: methods and protocols. 1st ed. Totowa, New Jersey: Humana Press; 2006. p. 53.

3. Wan H, Zhao Z, Qian C, Sui Y, Malik AA, Chen J. Selection of appropriate reference genes for gene expression studies by quantitative real-time polymerase chain reaction in cucumber. Anal Biochem. 2010 Apr 15;399(2):257-61. doi: 10.1016/j.ab.2009.12.008. Epub 2009 Dec 11. PMID: 20005862.

4. Kozera B, Rapacz M. Reference genes in real-time PCR. J Appl Genet. 2013 Nov;54(4):391-406. doi: 10.1007/s13353-013-0173-x. PMID: 24078518; PMCID: PMC3825189.

5. Hellemans J, Mortier G, De Paepe A, Speleman F, Vandesompele J. qBase relative quantification framework and software for management and automated analysis of real-time quantitative PCR data. Genome Biol. 2007;8(2):R19. doi: 10.1186/gb-2007-8-2-r19. PMID: 17291332; PMCID: PMC1852402.

6. D'haene B., Hellemans J. The importance of quality control during qPCR data analyis. Int. Drug Discov. 2010; 18–24.

7. Vandesompele J, De Preter K, Pattyn F, Poppe B, Van Roy N, De Paepe A, Speleman F. Accurate normalization of real-time quantitative RT-PCR data by geometric averaging of multiple internal control genes. Genome Biol. 2002 Jun 18;3(7):RESEARCH0034. doi: 10.1186/gb-2002-3-7-research0034. Epub 2002 Jun 18. PMID: 12184808; PMCID: PMC126239.

8. Pfaffl MW, Tichopad A, Prgomet C, Neuvians TP. Determination of stable housekeeping genes, differentially regulated target genes and sample integrity: BestKeeper--Excel-based tool using pair-wise correlations. Biotechnol Lett. 2004 Mar;26(6):509-15. doi: 10.1023/b:bile.0000019559.84305.47. PMID: 15127793.

9. Andersen CL, Jensen JL, Ørntoft TF. Normalization of real-time quantitative reverse transcription-PCR data: a model-based variance estimation approach to identify genes suited for normalization, applied to bladder and colon cancer data sets. Cancer Res. 2004 Aug 1;64(15):5245-50. doi: 10.1158/0008-5472.CAN-04-0496. PMID: 15289330.

10. Silver N, Best S, Jiang J, Thein SL. Selection of housekeeping genes for gene expression studies in human reticulocytes using real-time PCR. BMC Mol Biol. 2006 Oct 6;7:33. doi: 10.1186/1471-2199-7-33. PMID: 17026756; PMCID: PMC1609175.

11. Xie F, Xiao P, Chen D, Xu L, Zhang B. miRDeepFinder: a miRNA analysis tool for deep sequencing of plant small RNAs. Plant Mol Biol. 2012 Jan 31. doi: 10.1007/s11103-012-9885-2. Epub ahead of print. PMID: 22290409.
